# The prefrontal cortex as a target of HIV-1 neurotoxicity: molecular mechanisms of viral protein-mediated neurodegeneration and executive dysfunction

**DOI:** 10.3389/fncel.2026.1899622

**Published:** 2026-08-18

**Authors:** Erin S. Honsa

**Affiliations:** Department of Medical Education, Creighton University School of Medicine, Phoenix Campus, Phoenix, AZ, United States

**Keywords:** acquired immunodeficiency syndrome, AIDS, executive function, HAND, HIV, HIV-associated neurodegeneration, human immunodeficiency virus, prefrontal cortex

## Abstract

Despite the success of combined antiretroviral therapy (cART) in achieving peripheral viral suppression, HIV-associated neurocognitive disorder (HAND) remains a persistent challenge, affecting approximately 50% of people living with HIV. This review identifies the prefrontal cortex (PFC) as a primary target of HIV-1 neurotoxicity, as HIV-1 can directly infect the brain, and cART fails to eliminate the production of viral proteins from latent HIV-microglial reservoirs. The blood–brain barrier receives several hits from HIV-1 proteins, leading to a leaky and highly permeable barrier. Several proteins drive chronic neuroinflammation through inflammasome activation and pyroptosis, and ferroptosis in microglia. Glutamatergic dysregulation in the CNS results in increased neuron excitability and neurotoxicity. Also, overactive complement-mediated synaptic pruning and microglial senescence disrupt the intricate neural networks required for higher-order signaling. Neuroimaging and PFC dysfunction can be linked to specific HIV-1 proteins and their effects on the brain. Clinical symptoms of HAND directly reflect specialized PFC dysfunction. Deficits include impaired executive processes, which can present as lacking foresight for goal-directed actions, poor planning, and difficulty switching between tasks. Significant behavioral changes, including disinhibition, impaired moral reasoning, and increased risk-taking, can also be seen. Patients experience apathy, emotional blunting, and rapid mood shifts. Cognitive impairments include working memory loss, distractibility, and inappropriate memory linking. PFC disruptions can escalate into neuropsychiatric sequelae, driven by damaged dopamine and serotonin pathways. Defining these viral-mediated mechanisms will facilitate the development of pharmacological strategies that target several HIV-mediated neurotoxic pathways to prevent the debilitating characteristics of HAND.

## 1 Introduction

The central nervous system (CNS) relies on highly specialized interplay between diverse cell types and structural barriers to maintain the microenvironment required for complex neural signaling. Neurons serve as the fundamental functional units of the brain, using complex, intertwined networks to coordinate stimuli by receiving, processing, and transmitting signals (Maldonado and Alsayouri, 2026). Oligodendrocytes are critical cells that myelinate axons, providing insulation for efficient nerve conduction, help with neural plasticity, and maintain ion and water balance within the CNS (Simons and Nave, 2016). Within the neuronal network, microglia are essential resident phagocytes, performing caretaking duties such as regulating neurogenesis, clearing cellular debris, and pruning and shaping neural networks to maintain a healthy brain (Michell-Robinson et al., 2015). Astrocytes are responsible for supporting homeostasis, by controlling neurotransmitter levels and metabolic aid to promote neuron health, as well as providing physical support for the blood–brain barrier (BBB) (Gradisnik and Velnar, 2023). The BBB, an endothelial layer with extremely tight cell-to-cell connections, is a physical barrier that protects the CNS from toxic substances (Wu et al., 2023).

The prefrontal cortex (PFC) is a computationally and anatomically intricate brain region that serves as the primary orchestrator of autonomous, internally driven behavior (Chafee and Heilbronner, 2022). It is the location of higher-order cognitive processes, such as working memory, decision-making, planning, and behavior regulation. The PFC integrates instant-to-instant input from multiple cortical areas, comparing that information to past experiences to develop insight and foresight. Specific subareas handle specialized tasks: dorsolateral is associated with planning, strategy, and executive decisions (Hathaway and Newton, 2026). Dysfunction in this area results in deficits in working memory, cognitive flexibility, and executive function. These manifest in many ways, such as difficulty with task switching or dealing with rule changes, loss of working memory and subsequent inability to perform delayed response tasks, impairment in memory recall, and poor planning and lack of foresight for goal-directed actions. The orbitofrontal region is responsible for inhibiting primal survival responses that originate from the limbic system (Hathaway and Newton, 2026). Deficits here manifest as a loss of inhibitory and emotional control, and personality issues and decision-making impairment. Examples include socially inappropriate behavior, defective social or moral reasoning, increased risk-taking, behavioral disinhibition, rapid and inappropriate mood shifts, apathy and hypoactivity, confabulation, and distractibility (Hathaway and Newton, 2026). The PFC is critical for emotional regulation as it has extensive connections that control the release of neurotransmitters that effect mood: serotonin, dopamine, and norepinephrine. Disruption of the release of these chemicals can lead to neuropsychiatric disorders, such as depression, schizophrenia, and bipolar disorder (Hathaway and Newton, 2026).

The PFC also works as a top-down controller that monitors and regulates memory organization in the hippocampus, ensuring that related experiences are integrated while unrelated ones remain separated (de Sousa et al., 2026). When damaged, there is a significant disruption on how memories are separated, organized, and retrieved. This can be displayed as inappropriate memory linking, such as thinking that two events occurred simultaneously, even though they were a week apart.

Certain viruses, especially those that are neurotropic, represent a significant driver of PFC dysfunction as a complication of infection (Mohabbat and Bannazadeh Baghi, 2025; Srichawla et al., 2023; Davé and Klein, 2023). The damage is often multifaceted, including BBB disruption, peripheral cytokines entering the CNS, direct viral entry and infection of brain cells, and the ensuing neuroinflammatory response. Chronic, persistent signaling with proinflammatory cytokines, such as TNF-α, IL-1β, IL-6, IL-18, and interferons, cause widespread cellular dysfunction, damage, and cell death. The cumulative impact of molecular and cellular insults can cause PFC dysfunction, manifesting with the above-described executive deficits, memory problems, deficiencies in reward circuits, and personality changes. One of the most studied viruses that causes chronic neurological damage even with effective antiviral treatment is the Human Immunodeficiency Virus-1 (HIV-1) (Nightingale et al., 2023). This review summarizes the current knowledge on the mechanisms by which HIV-1 establishes and maintains infection in the PFC, and how viral proteins activate and dysregulate glial cells, induce neuronal damage and death, disrupt glutamatergic signaling, and ultimately impair PFC function. Understanding this intricate, complex set of viral-driven neurotoxicity can help elucidate novel targets to prevent, or reduce the severity of, HIV-associated neurodegenerative disorders (HAND) seen in people living with HIV (PLWH).

## 2 HIV-1 biology, neurotropism, and HAND

HIV was first discovered in 1983 as the cause of a newly identified disease, acquired immunodeficiency syndrome (AIDS) [Centers for Disease Control (CDC), 1981]. In the early 1980s, there was an alarming increase worldwide in the number of seemingly otherwise healthy young males developing infections, such as *Pneumocystis* pneumonia, and malignancies that were either previously unknown or associated with profound immunosuppression. Since the beginning of the AIDS epidemic, HIV has become one of the deadliest infectious agents of the modern scientific era, with estimates of over 44 million deaths since data collection began (World Health Organization, 2026). Developing countries continue to disproportionally experience the highest levels of HIV-associated morbidity and mortality, despite extraordinary scientific advances and the development of antiviral treatments. In 2024, there were 40 million PLWH worldwide, 31 million had access to antiretroviral therapy, and 630,000 died of HIV-related illness (Joint United Nations Programme on HIV/AIDS, 2025).

HIV-1, the most prevalent and virulent form worldwide, is a retrovirus that can integrate its genome into the host chromosome, establishing latent reservoirs that present a significant challenge to eradicate (Deeks et al., 2015). The virus is spread through several routes, with mucosal exposure constituting the most common route: sexual contact (with anal intercourse by far the highest risk), blood-borne and percutaneous routes, as well as vertically and postnatally through breastmilk (Shaw and Hunter, 2012). The most vulnerable populations for HIV infection continue to be men who have sex with men (MSM), injection drug users, Black/African American and Hispanic/Latino populations, displaced individuals, and sex workers (Joint United Nations Programme on HIV/AIDS, 2025). However, recent global trends indicate that in 2024, 45% of new HIV cases were among women and girls (Joint United Nations Programme on HIV/AIDS, 2025).

HIV has a unique replication cycle as a retrovirus, and multiple steps are targets for combined antiretroviral therapy (cART). After exposure, HIV primarily infects CD4^+^ T-cells via specific binding of viral surface glycoprotein, gp120, to the CD4 receptor (Engelman and Cherepanov, 2012). Once the virus enters, the viral reverse transcriptase converts the RNA genome into DNA, which is followed by genome integration into the host chromosome. This forms a provirus where viral proteins can be transcribed and translated, and mature virus released from infected cells. Cells expressing HIV co-receptors CCR5 and CXCR4 can also be infected, which include CNS cells of the monocyte/macrophage lineage, astrocytes, and oligodendrocytes. For a complete list of HIV cellular targets, see Coffin et al. (1997).

CD4^+^ T-cells play a central role in protective immunity by coordinating both innate and adaptive immune responses that prevent the development of infections and malignancies (including those caused by oncogenic viruses) (Luckheeram et al., 2012). CD4^+^ T cells play central roles in adaptive immunity by activating macrophages to eliminate intracellular pathogens, maintaining cytotoxic CD8^+^ T-cell antiviral and antitumor activity, and facilitating B-cell maturation and the production of high-affinity antibodies. A specialized subset, Th17 cells, help preserve epithelial barrier integrity, and help promote neutrophil defenses against extracellular bacteria and fungi.

During HIV infection, as CD4^+^ T-cells turn into virus-producing factories, their natural protective roles are lost. As their numbers decrease, there is an inverse increase in the development of opportunistic infections and malignancies (some known as AIDS-defining conditions), such as cryptococcal meningitis, toxoplasmosis, and non-Hodgkin lymphoma (Bento and Nguyen, 2026). AIDS is defined as a HIV-positive patient with a CD4^+^ level below 200 cells/mm^3^ (Battistini Garcia et al., 2026). These infections and malignancies are the traditional hallmark of late HIV infection.

**Figure 1:**
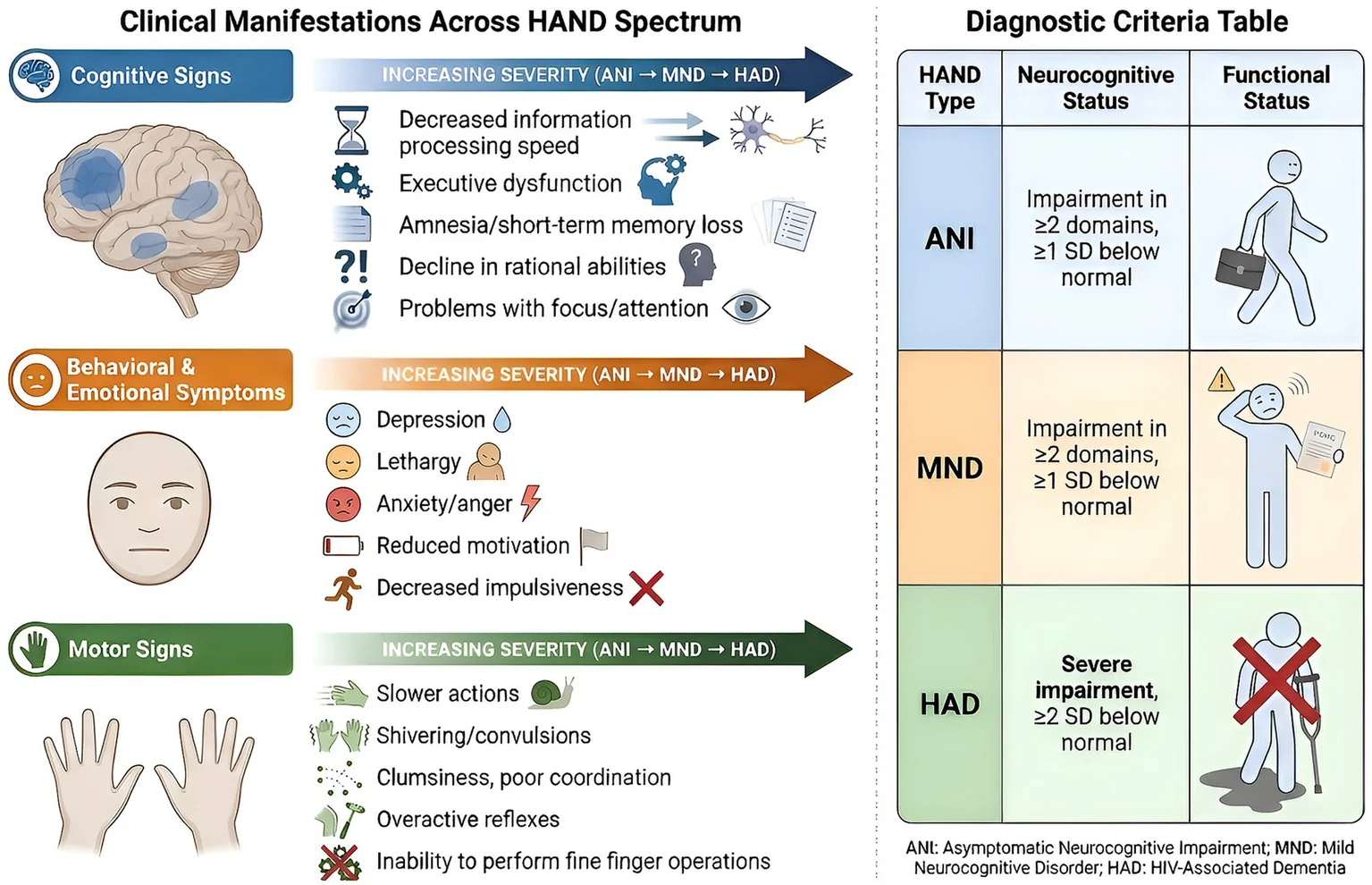
Clinical manifestations of HAND and diagnostic criteria **(Left)** Summary of the progressive cognitive, behavioral, and motor symptoms that characterize the escalation from asymptomatic neurocognitive impairment (ANI) to HIV-associated dementia (HAD). **(Right)** Specific diagnostic criteria for ANI, mild neurocognitive disorder (MND), and HAD, which are defined by the degree of neurocognitive impairment, measured in standard deviations (SD) below the mean, and the extent of interference with an individual’s daily functional status.

HAND represents a spectrum of neurological disorders that occur in up to 50% of PLWH during their lifetime, ranging from mild neuropsychiatric and neurocognitive impairments to more severe deficits, even when cART has successfully suppressed peripheral viral replication (Figure 1) (Wang et al., 2020). A clinical assessment is performed to help clinicians determine the neurocognitive impairment of their patients. It should be noted, however, that there is ongoing work to reach a single consensus to diagnose HAND (Nightingale et al., 2023; Gisslén et al., 2011). In general, the premise is that by testing patients with standardized neuropsychological testing (such as information processing, memory, and motor skills), they may be placed in one of three categories (Mitra and Sharman, 2026). Asymptomatic neurocognitive impairment (ANI) is when the patient scores one standard deviation or more below the mean (age-appropriate) in two or more cognitive domains, but do not demonstrate clear impairment. Mild neurocognitive disorder (MND) scores the same, but with evidence of functional impairment. The most severe form is HIV-associated dementia (HAD), with a score of two standard deviation or more below the mean in two or more cognitive domains, and evidence of functional impairment that interferes with daily living.

While cART has reduced the development of HAD, it has not eliminated HAND entirely (Eggers et al., 2017). Recent data suggests that in PLWH, 23% will develop ANI, 13% MND, and 5% HAD (Wang et al., 2020). Therefore, there is a multifaceted pathogenesis behind the development of HAND, driven by chronic inflammation triggered by the presence of HIV in the CNS, even when peripheral viral suppression has been achieved, representing an important aspect that is the focus of this review.

Evidence shows that soon after initial infection, peripheral monocytes are infected by HIV, and within the first 3 days, carry the virus through the BBB into the CNS via a Trojan Horse mechanism (Hazleton et al., 2010). HIV then quickly infects cells of the monocyte–macrophage lineage. Since microglia express surface receptors CCR5 and CXCR4, they are the primary CNS cells infected. Microglia have a long lifespan (a mean of 4.2 years) and divide slowly. Therefore, they represent a significant reservoir for HIV: not only for viral production, but subsequent chronic activation of CNS inflammation (Borrajo et al., 2021). In fact, as discussed below, there is overwhelming evidence that microglia are essential for the pathophysiology of HAND. Although HIV infects a subset of CNS cells, HIV proteins exert direct or indirect neurotoxic effects on other cell types, such as astrocytes, oligodendrocytes, and neurons. A plethora of evidence shows that during CNS HIV infection, proteins can either be secreted from infected microglial cells to interact with other cell types (bystander cells), or they are secreted within Extracellular Vesicles (EVs) or exosomes and diffuse within different brain compartments (Zhao et al., 2026; Tang et al., 2024). For example, HIV protein Vpr has been detected in the basal ganglia and PFC in patients with HIV-encephalopathy, and in transgenic mice expressing Vpr (Kogan and Rappaport, 2011). This caused neuronal damage in basal and subcortical areas, which is a result, in part, of microglial-mediated proinflammatory responses. Regardless of whether HIV proteins act directly on microglia, or are capable of intercellular communication, they have devastating consequences on neurodegeneration, employing overlapping mechanisms to support a chronic inflammatory environment within the brain parenchyma.

Questions that often arise when discussing HAND is if HIV can cross the BBB, why do half of PLWH never develop neurodegenerative symptoms? Why do some patients who are on effective long-term cART still develop HAND? Why do some patients only progress to ANI, while others will experience MND or HAD? Due to the nature of HIV infection, once a cell is infected, it exists either as an active viral production factory or persists in a proviral state where transcription can occur. Unfortunately, viral transcription (and thus translation of proteins) is not a target of cART, so neurotoxic proteins, especially early HIV proteins, may be expressed and secreted even if viral levels are undetectable. In fact, HIV RNA has been detected in the CSF of patients on cART who show no detectable serum viral load, suggesting that CNS production of virus may “escape” traditional cART suppression (Pérez-Valero et al., 2019). Evidence shows that in a subset of PLWH, a higher rate of provirus transcription is established (Lichterfeld et al., 2022). To further add insult to injury, certain antiretroviral drugs demonstrate poor CNS penetration, such as tenofovir and some protease inhibitors, reducing the effective viral suppression capabilities (Ene et al., 2011). Finally, certain individuals may have higher “baseline” microglial activation, potentiating an increased chance of neuroinflammation and subsequent development of neurodegenerative diseases (Schaffer Aguzzoli et al., 2023).

## 3 The pathophysiological effects of HIV-1 on the CNS

Since HIV-1 infects microglial cells early in infection, it is not surprising that many viral components activate these CNS immune cells, resulting in chronic proinflammatory processes and resulting in a neurotoxic state. The ability of several HIV proteins to target other CNS cells once released from microglia further contributes to a dysfunctional state that can cause neuron injury or death. As mentioned, since cART does not eliminate the provirus nor inhibit transcription/translation of viral products, this inflammatory state can occur even in individuals with no detectable peripheral virus. In fact, several HIV proteins have been detected in the brains of PLWH on cART (Donoso et al., 2022). Due to the focus of this review, please see Zhao et al. (2026) for an extensive in-depth summary of the current understanding of the effects of HIV viral RNA on microglia.

Before discussing the proinflammatory effects of HIV proteins, it is important to understand their normal role in HIV infection and replication [German Advisory Committee Blood (Arbeitskreis Blut), Subgroup ‘Assessment of Pathogens Transmissible by Blood’, 2016]. Gp120 is the main surface glycoprotein of HIV that mediates attachment to CD4 receptor and co-receptors CCR5/CXCR4 (Kwong et al., 1998). Gp41 is a transmembrane protein that helps gp120 securely anchor to cell receptors, and aids in cell membrane fusion. Trans-activator of transcription (Tat) is an essential early protein of HIV that regulates and increases the rate of viral transcription (Faust et al., 2017). Negative regulatory factor (Nef) is an early accessory protein that is important for immune evasion, such as downregulating MHC-1 in infected cells to reduce cytotoxic T-cell recognition, and aids in high-level viral replication by downregulating CD4 receptor expression which prevents “superinfection” of T-cells and premature death (Lundquist et al., 2002). Group-specific antigen (Gag) is a multiprotein transcript which encodes, in part, the structural proteins for the capsid (p24) and matrix (p17) (Klingler et al., 2020). Viral protein r (Vpr) is essential for HIV replication in macrophages and resting T-cells and induces cell cycle arrest to optimize the intracellular environment for viral transcription (Li et al., 2005). While this ultimately leads to cell death via apoptosis, it provides an efficient provirus state while the infected host cells are alive. Viral protein unique (Vpu) is an accessory protein that is important during the late stage of viral replication, as it helps induce the degradation of new CD4 receptors in infected cells, which would otherwise interfere with viral envelope processing and budding.

HIV protein expression occurs even in the presence of cART and can affect the infected cell directly. Also, research shows that certain HIV proteins can be packaged inside EVs. These are nano-sized particles derived from microglia cells (in context of this review) that allow for intercellular communication within the brain. Evidence shows that if EVs contain a pathogen-associated molecular pattern (PAMP), the recipient microglia can be activated as if it was exposed to the PAMP alone (Pérez et al., 2019). In theory, this would allow a sensitive and directed proinflammatory response. During CNS infection (neuroHIV), EVs are released containing Tat and Nef, which are transcribed and translated regardless of cART or viral suppression (Zhao et al., 2026). Therefore, even non-HIV infected microglia and other CNS cells may be affected. As will be discussed, EV-Tat causes the shortening of neurons and their death by an NLRP3 inflammasome-mediated pathway (Kannan et al., 2022). EV-Nef has been shown to impair the BBB, induce oxidative stress within the brain, and activate the inflammasome in microglia (Mukhamedova et al., 2019). This can lead to axonal degeneration and an Alzheimer’s-like pathophysiology, which is one of the hallmarks of HAD (Zhao et al., 2026). Together, the presence of HIV proteins throughout the brain collectively exerts detrimental effects on neuronal health and PFC function, resulting in subsequent neurocognitive disorders discussed in this review. The following subsections will detail the specific roles and pathogenesis of HIV proteins in establishing a proinflammatory state, microglial dysfunction, direct neurotoxicity, and BBB impairment that together contribute to HAND. Table 1 summarizes the roles of important HIV proteins in these outcomes.

**Table 1:** Direct roles and effects of HIV proteins in the CNS.

HIV Protein	Molecular role/mechanism	Cellular and molecular effects	Downstream CNS impact	References
gp120	Binding to CXCR4/CCR5 receptors on microglia	Activates NF-κB signaling, upregulating transcription of NLRP3 and pro-IL-1B (priming); triggers K^+^ efflux (activation)	NLRP3 inflammasome assembly and activation	He et al. (2020)
	Binding to CCR5 on BMECs for direct viral infection	Cytoskeletal alterations, ROS increase leads to increased expression of MMPs that degrade TJ proteins	Highly permeable and leaky BBB	Annunziata et al. (1998); Cioni and Annunziata (2002); Kanmogne et al. (2007); Shiu et al. (2007); Louboutin and Strayer (2012)
	Induction of iron dysregulation	Increases free redox-reactive intracellular Fe^2+^ by causing endolysosomal deacidification and iron efflux into the cytoplasm	Ferroptosis in microglia	Khan et al. (2021); Halcrow et al. (2022)
	Promotion of oxidative stress	Increases ROS levels and promotes lipid peroxidation	Amplifies proinflammatory pathways; indirect neuronal death through redox imbalance; increases likelihood of ferroptosis	Zhao et al. (2026)
	Upregulation of complement components C1q and C3; activate astrocytes to secrete more C3	Increases complement deposition on synapses	Aberrant and overzealous synaptic pruning by microglia	Watson et al. (2025); Nitkiewicz et al. (2017)
	Binds to NMDARs on neurons	Triggers Ca^2+^ influx into cytoplasm	Causes neuronal depolarization, mitochondrial instability, and loss of presynaptic terminals	Gemignani et al. (2000); Liu et al. (2023)
	Reduces expression of EAAT2 and glutamine synthase in astrocytes	Reduces astrocyte ability to import and neutralize excess extracellular glutamate in the CNS	Disruption of glutamatergic signaling due to build-up of excitatory glutamate, leading to neuron overexcitability	Wang et al. (2003); Chen et al. (2023)
gp41	Direct activation of the NAIP/NLRC4 inflammasome	Binds directly to sensor NAIP in microglia	NLRC4 inflammasome assembly and activation	Triantafilou et al. (2021)
	Induction of NOS	Increases production of NO	Elevated NO toxic to microglia and neurons	Hu et al. (1999)
Nef	Reduces cholesterol efflux in microglia	Alters membrane location of TLR4, causing activation and signaling cascade	NLRP3 inflammasome assembly and activation	Mukhamedova et al. (2019)
	Activates NADPH oxidase in microglia	Triggers signaling cascades that induce transcription of proinflammatory mediators	Microglia release CCL5/RANTES, IL-6, IL-8, and IL-12	Si et al. (2002)
	BBB disruption	Reduces TEER; causes downregulation of ZO1	Highly permeable and leaky BBB	Raymond et al. (2016)
	Directly damages oligodendrocytes	Decrease in mature oligodendrocyte populations	Loss of myelinated axons	Schenck et al. (2024)
Tat	Decreases mitophagy in microglia	Damages mitochondria, causing their dysfunction	Increases oxidative stress in microglia	Thangaraj et al. (2018)
	Increases intracellular Ca^2+^, causing mitochondrial stress and mtDNA leakage	Both Ca^2+^ and mtDNA are DAMPs that trigger inflammasome formation	NLRP3 inflammasome assembly and activation	Zhao et al. (2026); Rossol et al. (2012); Yang et al. (2025)
	Induction of iron dysregulation	Increases free redox-reactive intracellular Fe^2+^	Ferroptosis in microglia	Khan et al. (2021); Halcrow et al. (2022)
	Activates ACSL4 in microglia	Enriches cell membranes with oxidizable lipids		Kannan et al. (2023)
	Suppresses antioxidant enzyme GPX4	Loss of ability to neutralize oxidized lipids		Li and Chen (2026)
	Upregulation of complement components C1q and C3; activate astrocytes to secrete more C3	Increases complement deposition on synapses	Aberrant and overzealous synaptic pruning by microglia	Lu et al. (2011); Nitkiewicz et al. (2017)
	Increases expression of p16 and p21	Cell cycle arrest	Microglial senescence	Thangaraj et al. (2020)
	Causes downregulation of CXC3R1 (FKN receptor) on microglia	Microglia less efficient at responding to fractalkine	Dysregulation of calcium signaling leads to chronic microglial activation	Duan et al. (2014); Hasan et al. (2024); Niswender and Conn (2010)
	Binds to NMDARs on neurons	Triggers Ca^2+^ influx into cytoplasm	Causes neuronal depolarization, mitochondrial instability, and loss of presynaptic terminals	Duffy et al. (2026); Self et al. (2004)
	Interacts with AMPARs on oligodendrocytes	Triggers Ca^2+^ influx into cytoplasm	Causes cell dysfunction and death, leading to loss of myelin sheaths	Zou et al. (2015)
	Interacts with mGluRs on neurons	Causes increased release of glutamate	Synaptic imbalance	Musante et al. (2010); New et al. (1998)
	Reduces expression of EAAT2 and glutamine synthase in astrocytes	Reduces astrocyte ability to import and neutralize excess extracellular glutamate in the CNS	Disruption of glutamatergic signaling due to build-up of excitatory glutamate, leading to neuron overexcitability	Duffy et al. (2026); Ye et al. (2017)
	Upregulation of cystine-glutamate antiporter in microglia and astrocytes	Causes increased release of glutamate	Synaptic imbalance	Marino et al. (2020); Gupta et al. (2010)
	BBB disruption	Causes downregulation of claudin-1, claudin-5, and ZO2	Highly permeable and leaky BBB	András et al. (2003); Leibrand et al. (2017)
	Induction of NADPH oxidase and NOS in microglia	Increases production of NO	Elevated NO toxic to microglia and neurons	Polazzi et al. (1999)
Vpr	Damages mitochondria and causes oxidative stress	Creates DAMPs that trigger inflammasome formation	NLRP3 inflammasome assembly and activation	Mamik et al. (2017)
	Activation of HIF-1	Induces production of H2O2	ROS-triggered inflammation	Deshmane et al. (2009)
	Binds to mitochondrial membrane in neurons	Reduces ATP production, increases ROS, disrupts mitochondrial transport	Neuronal death	Kitayama et al. (2008)
	Arrests neuronal cell cycle at G2/M phase	Caspase-3, −7, and −8 activation		Andersen et al. (2006); Patel et al. (2000)
	Activates signaling pathways in both microglia and astrocytes	Induces RANTES production	Recruitment of peripheral T-cells and monocytes into brain	Si et al. (2002)
Vpu	Binds to TLR4 on microglia	Triggers transcription of pro-IL-1B and NLRP3	Primes the NLRP3 inflammasome	Triantafilou et al. (2021); Xu et al. (2020)
	Interacts with K^+^ channels in cell membrane	Causes K^+^ efflux, a DAMP	NLRP3 inflammasome assembly and activation	Xu et al. (2020)

### 3.1 Chronic proinflammatory responses activated by HIV in microglia

It is not surprising that many HIV proteins trigger the production of proinflammatory cytokines by a plethora of signaling cascades that rely on molecular factors such as NF-κB to induce transcription. For general reactive oxygen species (ROS)-triggered inflammation in microglia, ROS act as signal molecules that activate redox-sensitive pathways such as NF-κB and MAPK, leading to secretion of proinflammatory cytokines TNF-α, IL-1β, IL-6, and IL-8 (Simpson and Oliver, 2020). Several HIV proteins have been shown to increase ROS levels and subsequent stress by a number of pathways, including the role of Tat in decreasing mitophagy in microglia, leading to damaged and dysfunctional mitochondria that increases oxidative stress (Thangaraj et al., 2018). Vpr has been shown to trigger H_2_O_2_ production by activation of hypoxia-inducible factor 1 (HIF-1) (Deshmane et al., 2009). In fact, in PLWH with HAD, HIF-1 was notably elevated (Deshmane et al., 2009). Nef can activate NADPH oxidase in microglia, leading to neuroinflammation (Vilhardt et al., 2002). It has also been shown that when human microglial cells were treated with Nef secreted from Nef-transduced microglia (mimicking secreted Nef during natural HIV infection), they secreted significant levels of CCL5/RANTES, IL-6, IL-8, and IL-12 (Raymond et al., 2016). Tat, gp41, and gp120, can induce the expression of nitric oxide (NO) synthase, leading to elevated NO levels that can damage microglia and surrounding neurons (Polazzi et al., 1999; Hu et al., 1999; Walsh et al., 2004). Tat has been shown in studies using human monocytes and dendritic cells to interact with, and activate, TLR4 signaling, resulting in an overproduction of TNF-*α*, IL-6, IL-8, and IL-10 (Haij et al., 2015; Ben Haij et al., 2013). It can also interact with the MyD88 and TRIF pathways to further enhance proinflammatory responses in myeloid cells (Planès et al., 2016). Finally, Vpr and Nef have both been shown to activate pathways in microglia (and astrocytes) that cause RANTES production (Si et al., 2002). This helps with the recruitment of T-cells, monocytes, and eosinophils, potentiating a strong, chronic inflammatory state. RANTES upregulation in the brain has been implicated not only with HIV-driven neurodegeneration, but other conditions such as Parkinson’s disease (Tang et al., 2014).

### 3.2 Microglial activation via NLRP3 inflammasome and pyroptosis

**Figure 2:**
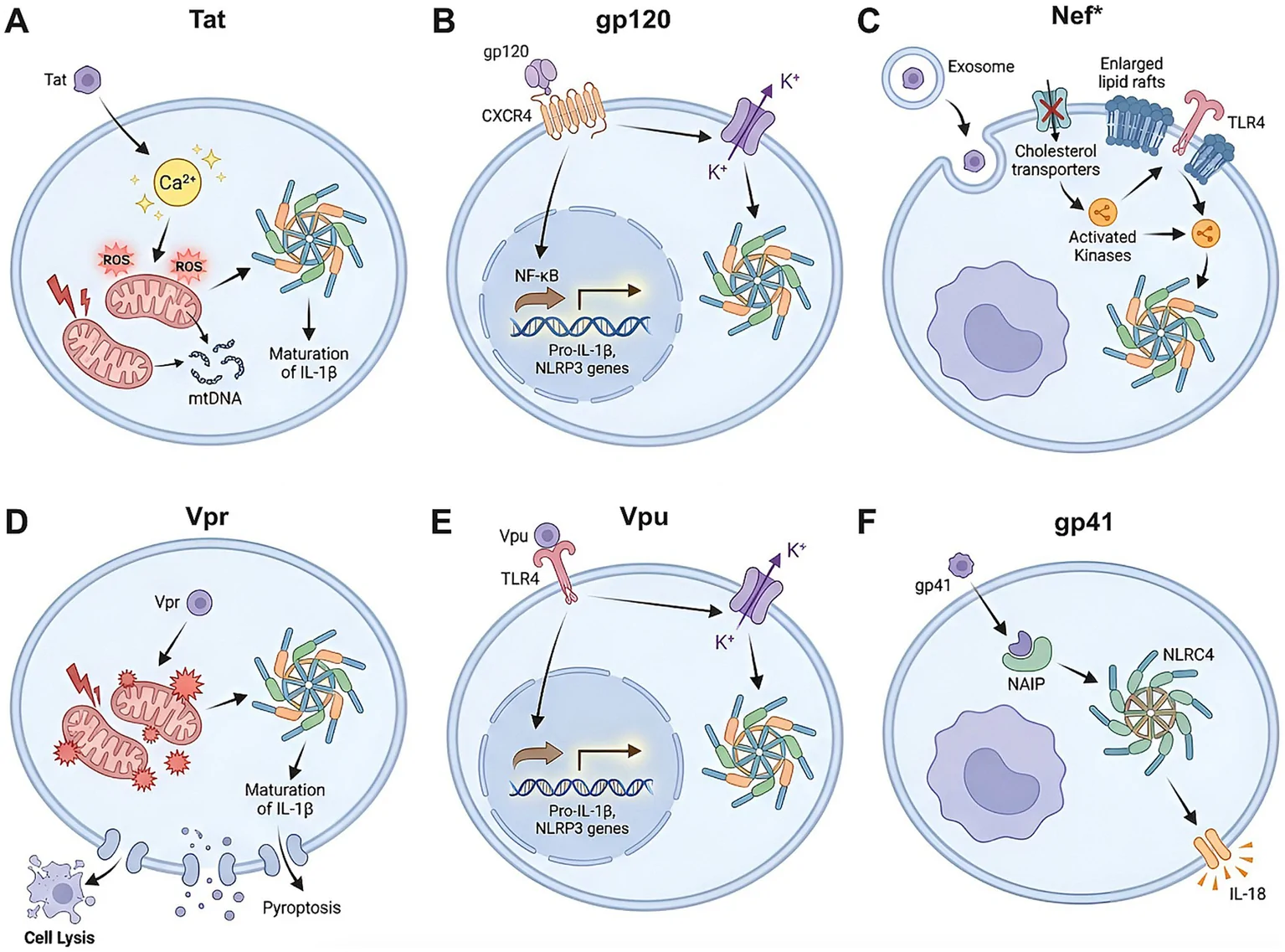
Mechanisms of inflammasome activation by HIV-1 proteins. HIV-1 proteins activate multiple inflammasome signaling pathways through distinct but overlapping mechanisms in microglia, causing pyroptosis and release of pro-inflammatory cytokines IL-1β and IL-18. Tat induces intracellular calcium influx, mitochondrial dysfunction, reactive oxygen species (ROS) production, and mitochondrial DNA (mtDNA) release, leading to NLRP3 inflammasome (blue, orange, green multi-protein unit) activation, caspase-1-mediated maturation of IL-1β, and amplification of neuroinflammation **(A)**. Gp120 engages chemokine receptors CXCR4, promoting NF-κB-dependent transcriptional priming of inflammasome components and potassium (K^+^) efflux, a key signal for NLRP3 inflammasome assembly **(B)**. Nef* (exosome-Nef) disrupts cholesterol homeostasis by inhibiting cholesterol transporters, enlarges lipid rafts, activates Toll-like receptor 4 (TLR4, pink membrane protein)-dependent kinase (orange circles) signaling, and promotes NLRP3 inflammasome activation **(C)**. Vpr induces mitochondrial stress and oxidative damage, resulting in inflammasome activation, IL-1β maturation **(D)**. Vpu activates TLR4 signaling and promotes K^+^ efflux, providing both priming and activation signals for NLRP3 inflammasome assembly and pro-inflammatory cytokine production **(E)**. Gp41 activates the NAIP (green)/NLRC4 inflammasome (blue, green, orange pinwheel) pathway, leading to caspase-1 activation and IL-18 secretion **(F)**.

The inflammasome requires a signal to be detected within infected or damaged cells, which in turn activates caspase-1 (Zheng et al., 2020). This results in the secretion of active proinflammatory cytokines IL-1β and IL-18 during pyroptosis. There is strong evidence that following HIV-1 infection of microglia, caspase-1 and NLRP3 protein levels are increased, suggesting a priming response to the presence of HIV (Table 1; Figure 2; Walsh et al., 2014). Tat has been extensively researched and noted to be a strong contributor to inflammasome formation via its effects on microglial cells (Figure 2A). Firstly, Tat is able to increase intracellular Ca^2+^ within microglia, which is a known danger-associated molecular pattern (DAMP) signal for inflammasome activation (Zhao et al., 2026; Rossol et al., 2012). Secondly, Tat was recently discovered to cause neurotoxicity by inducing mitochondrial stress and increase in ROS, leading to leakage of mitochondrial DNA (mtDNA) into the cytoplasm, another inflammasome activation DAMP signal (Yang et al., 2025).

Gp120 can cause the priming and subsequent activation of the NLRP3 inflammasome, albeit by a different mechanism (Figure 2B). When gp120 binds to CXCR4 on microglial cells, receptor signaling activates NF-κB, which in turn upregulates the transcription of NLRP3 and pro-IL-1β (He et al., 2020). This primes the cell for inflammasome assembly. Gp120 can also trigger K^+^ efflux when binding to the cell co-receptor, causing the direct activation of the NLRP3 inflammasome, caspase-1-mediated activation of pro-IL-1β, and subsequent surrounding inflammatory-driven neuronal dysfunction and death.

Nef is also responsible for the activation of the NLRP3 inflammasome in microglia (Figure 2C). Research shows that once macrophages were exposed to Nef in exosomes, cholesterol efflux was reduced, which resulted in an increase in lipid rafts in the membrane (Mukhamedova et al., 2019). This altered the localization of TLR4, activating signaling kinases, and subsequent primed and activated the NLRP3 inflammasome, leading to a strong proinflammatory response.

Vpr has been detected in the CSF of patients with HIV, and in supernatants of HIV-infected human microglia (Figure 2D; Kogan and Rappaport, 2011). This protein was shown to directly promote the production of IL-1β in primary human microglia, in an NLRP3-dependent manner, activating the assembly of the inflammasome and subsequently caused reduced cell viability due to pyroptosis (Mamik et al., 2017). This study also observed that transgenic mice who expressed Vpr in the brain developed neurotoxicity, with resulting neurodegeneration typically associated with HAND. The ability of Vpr to damage mitochondria and cause oxidative stress provides the DAMP signal that initiates NLRP3 activation.

Vpu can prime the inflammasome pathway via binding to TLR4, which results in transcription of pro-IL-1β and NLRP3 (Figure 2E; Triantafilou et al., 2021). There is also evidence that Vpu interacts with cell membrane K^+^ channels, causing K^+^ efflux. This reduction in cytosolic K^+^ is a trigger (DAMP) for NLRP3 activation, by causing a conformational change and assembly of the multi-protein inflammasome unit (Xu et al., 2020).

It should be noted that recent evidence has shown that gp41 can directly activate the NAIP/NLRC4 inflammasome without any priming, leading to secretion of active IL-18 (Figure 2F). This is due to the ability of gp41 to bind to the pathway’s sensor protein, NAIP, in macrophages (Triantafilou et al., 2021).

Together, several components of HIV can prime and activate the inflammasome, which facilitates the production of IL-1β and IL-18 during pyroptosis. This induces strong proinflammatory neurotoxic effects, such as increasing the permeability of the BBB, that is amplified by several other microglial activation events or dysfunctional states described below, as well as subsequent amplification of other inflammatory responses, cell damage, excitotoxicity, and death of neurons.

### 3.3 Ferroptosis leads to microglial dysfunction and death

**Figure 3:**
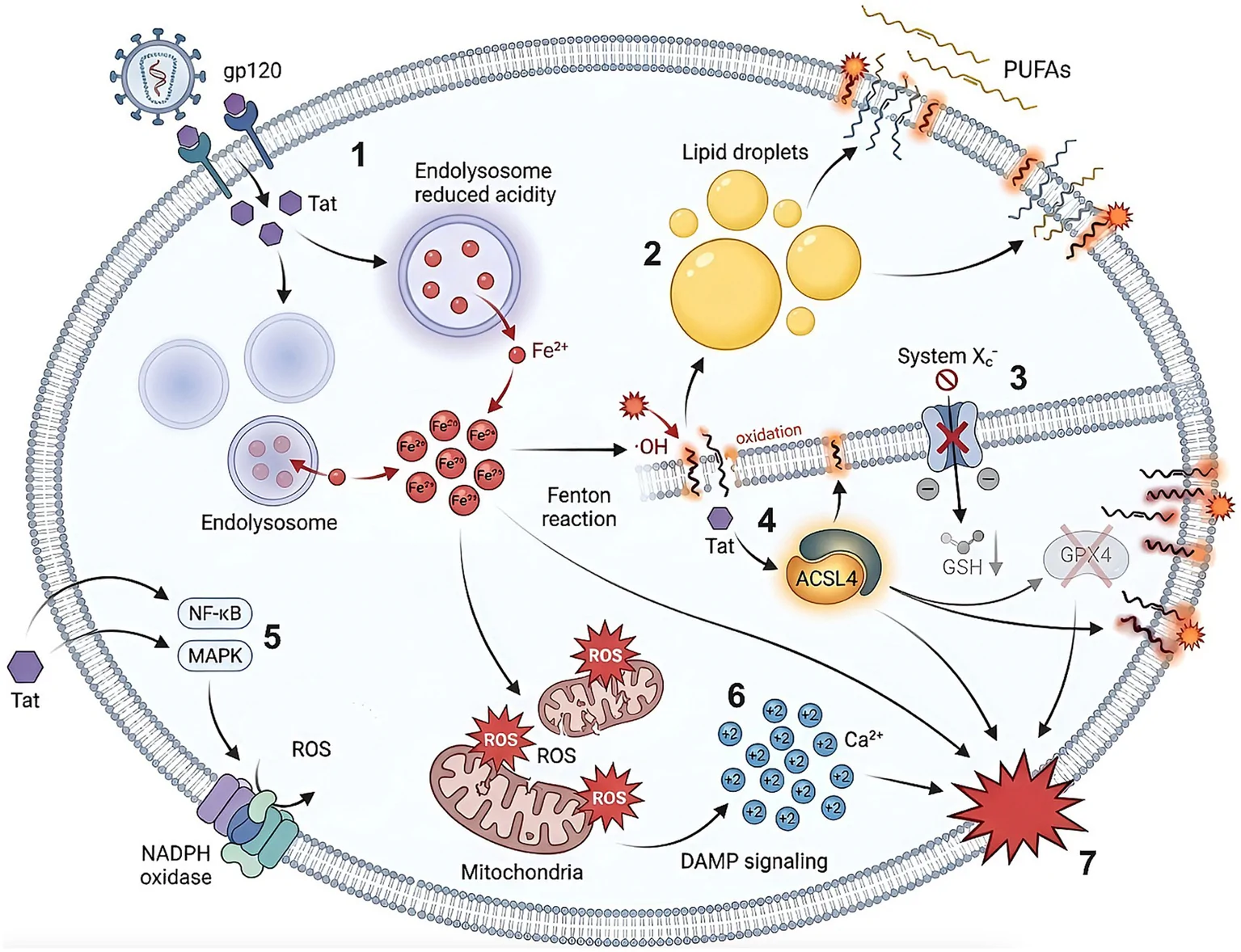
HIV-1 activates ferroptosis in microglia by several mechanisms. HIV proteins Tat and gp120 (purple hexagons) induce ferroptosis through coordinated disruption of iron homeostasis, antioxidant defenses, lipid metabolism, and mitochondrial function. 1. Tat and gp120 impair endolysosomal acidification, promoting lysosomal iron release and expansion of the labile intracellular Fe^2+^ pool. Ferrous iron catalyzes the Fenton reaction, generating highly reactive hydroxyl radicals (•OH, orange starburst) that initiate lipid peroxidation. 2. Excess iron promotes lipid droplet accumulation and oxidation of membrane phospholipids containing polyunsaturated fatty acids (PUFAs), increasing susceptibility to ferroptotic membrane damage (red and orange membrane lipids). 3. Tat inhibits the cystine/glutamate antiporter (system Xc^−^) which reduces intracellular cystine uptake, leading to glutathione (GSH) depletion and impaired glutathione peroxidase 4 (GPX4) activity, thereby preventing detoxification of lipid hydroperoxides (red and orange membrane lipids). 4. Tat enhances expression and activity of acyl-CoA synthetase long-chain family member 4 (ACSL4), increasing incorporation of PUFAs into membrane phospholipids and promoting lipid peroxidation. 5. Tat also activates NF-κB- and MAPK-dependent signaling pathways, stimulating NADPH oxidase (purple, blue, and green membrane protein) activity and increasing reactive oxygen species (ROS) production. 6. HIV proteins induce mitochondrial dysfunction, resulting in excessive mitochondrial ROS generation, calcium dysregulation, and release of damage-associated molecular patterns (DAMPs), which further amplify oxidative stress. Collectively, these processes drive overwhelming lipid peroxidation, membrane disruption, and 7. ferroptotic cell death through membrane rupture.

Ferroptosis is a newer form of proinflammatory, iron-dependent cell death officially named in 2012 (Dixon et al., 2012). This non-apoptotic death pathway has been implicated in several diseases, including cancers, liver fibrosis, and neurodegenerative disorders. This “sterile” response is triggered by several DAMPS, including Ca^2+^ and accumulation of ROS. The overall intracellular state includes a combination of free iron overload, compromised antioxidant systems and subsequent buildup of ROS, and the increase in oxidizable lipids (polyunsaturated fatty acids, PUFAs) in the cell membrane (Li et al., 2020). In general, when a cell is depleted of glutathione (GSH), the activity of glutathione peroxidase 4 (GPX4) is compromised, and lipid peroxides can no longer be neutralized. As free Fe^2+^ builds, the Fenton reaction oxidizes membrane PUFAs. Once these lipids are peroxidized, the membrane loses its integrity, breaks apart, and the cell dies. In the CNS, microglia are the most iron-rich cells, resulting in sensitivity to ferroptosis if any one of the regulatory pathways to prevent its activation is compromised (Jiao et al., 2022). Several studies have demonstrated that Tat (and in part, gp120) are key mediators of ferroptosis activation by interfering with multiple events that all lead to death and release of proinflammatory cytokine (Figure 3; Zhao et al., 2026). This creates a neuroinflammatory feedback loop, and amplification of other proinflammatory pathways that have a role in neurodegeneration, such as excessive synaptic pruning.

Ferroptosis is reliant on iron to activate lipid peroxidation. Tat and gp120 have been shown to increase the pool of free redox-reactive intracellular Fe^2+^ by causing endolysosomal deacidification during initial viral infection, which causes efflux of free iron into the cytoplasm (Khan et al., 2021; Halcrow et al., 2022). This increases the opportunity for Fenton reactions to attack lipids. Secondly, Tat, and general proinflammatory activation of microglia by HIV, release lipid droplets via lipid dysregulation (Cheng et al., 2025). These droplets help traffic and incorporate PUFAs into the cell membrane, which act as the prime target for peroxidation. Therefore, the overall target lipid pool increases. Thirdly, Tat suppresses an important antioxidant enzyme, GPX4, by inhibiting cystine import (System Xc^−^) and GSH synthesis, preventing GPX4 function (Li and Chen, 2026). This results in unchecked accumulation of even more lipid peroxides, as Fe^2+^ attacks the PUFAs in the plasma membrane. Fourthly, Tat specifically activates Acyl-CoA Synthase Long-Chain Family Member 4 (ACSL4), a “Master Regulator” of ferroptosis activation (Kannan et al., 2023). When ACSL4 is active, PUFAs are enriched in the cell membrane, further amplifying available lipids to be peroxidized to levels that trigger ferroptosis. Fifthly, as discussed above, Tat activates NADPH oxidase in microglia via NF-κB and MAPK signaling, which causes mitochondrial damage and ROS leakage, which increases lipid peroxidation. Finally, Tat increases intracellular Ca^2+^ within microglia, a known DAMP trigger for ferroptosis, as it increases intracellular ROS (Sheng et al., 2000). In summary, Tat increases intracellular free iron, ROS, and PUFAs in the cell membrane, triggering ferroptosis and release of proinflammatory cytokines from dead microglia. This occurs in both infected microglia, and bystander cells that are “infected” with EVs containing Tat (Zhao et al., 2026). Even if microglia do not immediately die, iron-activated cells demonstrate increased atypical synaptic pruning (Sfera et al., 2022). Another unfortunate result of HIV and its role in triggering ferroptosis and generalized neuroinflammation, is cytokines such as TNF-α can affect neighboring uninfected neurons by increasing internal ROS stress, disrupt their redox balance, and generate signals that trigger neuron death.

### 3.4 Excessive active synaptic pruning by microglia

Synaptic pruning in the CNS is mediated, in part, by complement factors C1q (classic pathway) and C3 (alternative pathway) (Gomez-Arboledas et al., 2021). Studies have shown that in HIV infection, even in patients with undetectable viral production, C1q and C3 levels are increased in the cortex, which correlated with dysregulated microglial pruning (McGuire et al., 2016). Imaging studies also revealed a global decrease of connectivity between brain regions in PLWH that were diagnosed with any severity of HAND, not just HAD. In fact, there is a correlation between loss of excitatory synaptic density and HIV-related neurodegenerative impairment (Everall et al., 1999).

HIV proteins Tat and gp120 have been implicated in the increased expression of these complement components, in both mice studies and in the brain tissue of PLWH demonstrating cognitive impairment. In mice studies, injection of Tat into the PFC caused microglial activation, peripheral monocyte infiltration (leading to secretion of C1q), and subsequent loss of synapses (Lu et al., 2011). Further, transgenic mice expressing gp120 in the PFC had a significant increase of synaptic pruning of both excitatory and inhibitory synapses (Watson et al., 2025). This same study also demonstrated increased synaptic engulfment around areas of neuroinflammation in the brain tissue of PLWH. Together, the current literature suggests that Tat and gp120 are the main drivers of non-autonomous microglial synaptic pruning, leading to excessive neuronal loss. So how do these HIV proteins and complement cause aberrant microglial pruning?

As noted earlier, Tat and gp120 activate microglia via several pathways that result in the secretion of proinflammatory cytokines. Focusing on their downstream effects of increasing complement activator components in the CNS, it is not surprising that these cytokines directly activate astrocytes. Therefore, astrocytes indirectly respond to the presence of HIV-induced inflammation by increasing the expression and secretion of C3 through NF-κB activity (Nitkiewicz et al., 2017). The abundance of C3 within neuroinflammatory sites, and the presence of active microglia searching for the C3 “eat me” signal that is aberrantly deposited on neuron synapses, leads to their inappropriate destruction. It should be noted that synaptic dysfunction has been determined in neuroHIV (McLaurin et al., 2019). This correlates with executive function and memory deficiencies, a hallmark of PFC dense synaptic connectivity disruption.

### 3.5 Microglial senescence

While microglial activation and complement-mediated phagocytosis have been shown to have a direct role in the pathophysiology of HAND, HIV-triggered microglial senescence also seems to be a contributing element (Zhao et al., 2026; Mason et al., 2025). This phenomenon has been identified in both HIV-infected and bystander microglial cells, suggesting a global CNS effect on normal microglial function. Senescence has traditionally been implicated in aging and diseases such as Alzheimer’s; however, growing evidence shows a strong link between HIV neurotoxicity, microglial senescence, and HAND (Zhao et al., 2026; Malvaso et al., 2023). HIV Tat is the main driver behind this dysfunctional state, causing premature senescence of microglia which results in mitochondrial dysfunction (and subsequent oxidative stress), leading to proinflammatory cytokine release. Tat also reduces proliferation capacity by promoting cell cycle arrest by increasing the expression of p16 and p21 (Thangaraj et al., 2020). So, while activation of pyroptosis and ferroptosis in microglia by HIV may trigger cell death, Tat can also prevent cell proliferation and increase cell life expectancy, along with a sustained proinflammatory state. Together, the induction of senescence by HIV produces aging microglia that are highly proinflammatory and overzealous in their phagocytic hyper-pruning activities (amplifying complement-mediated pruning dysfunction).

### 3.6 Disruption of the CX3CL1-CX3CR1 signaling axis

As part of inflammation homeostasis within the CNS, neurons constitutively express fractalkine (CX3CL1), which binds to the FKN receptor (CX3CR1) expressed on the surface of microglia (Harrison et al., 1998; Mattison et al., 2013; Cardona et al., 2006). This axis is responsible, in part, for regulating complex interactions between the two cell types, resulting in reduction of microglial-mediated neurotoxicity and subsequent neurodegenerative disorders (Cardona et al., 2006). The chemokine has also been shown to protect neurons against glutamate-mediated excitotoxicity (Limatola et al., 2005). Several studies have shown that deficiencies in FKN receptor expression in mice resulted in excessive proinflammatory production by microglia, decreased survival of neurons, and subsequent impaired cognitive function (Bachstetter et al., 2011; Rogers et al., 2011).

As discussed thus far in this review, HIV proteins cause a dysregulation of several arms of CNS inflammation, as well as reducing the number of neurons available to secrete fractalkine. A study by Duan et al. (2014) discovered that Tat specifically disrupted the CX3CL1-CX3CR1 signaling axis during HIV-1 infection by downregulating the expression of the FKN receptor. When Tat is expressed within microglia, or internalized from external sources, a specific molecular cascade is triggered, which resulted in less receptor expression. Specifically, Tat activates NF-κB, which in turn increases the expression of YY1, a transcriptional repressor in microglial cells. Once this occurs, YY1 binds to a specific consensus sequence within microglial DNA, silencing the transcription of *CX3CR1* in both rat and human cells (Duan et al., 2014). This caused a reduction of both mRNA and protein levels, rendering microglia less efficient at responding to fractalkine.

The resulting loss of FKN receptor expression profoundly impairs the functional responsiveness of microglia to neuronal-derived fractalkine, leading to diminished internal calcium mobilization and microglial migration. Dysregulation of calcium signaling leads to chronic activation, by preventing normal quenching of microglial activity (Hasan et al., 2024). This results in the increased induction and release of increased release of neurotoxic cytokines such as TNF-α, IL-1β, IL-6, and CXCL10 from microglia, subsequently contributing to the pathogenesis of HAND (Duan et al., 2014). By undermining the reciprocal communication between neurons and microglia, Tat creates a state of protracted microglial activation and neuroinflammation that contributes to exacerbated CNS pathology. The resulting loss of supportive microglial functions, combined with the continuous release of neurotoxins, underlies the cognitive, motor, and behavioral impairments observed in HAND patients. Thus, the Tat-mediated disruption of the microglial CX3CL1-CX3CR1 axis serves as a key driver of chronic neuroinflammation and CNS dysfunction in the setting of HIV infection.

### 3.7 HIV-1-mediated glutamatergic dysfunction

Glutamate is the primary excitatory neurotransmitter in the CNS, binding to *α*-amino-3-hydroxy-5-methyl-4-isoxazolepropionic acid (AMPA) receptors (AMPAR) for rapid initial excitation, N-methyl-D-aspartate (NMDA) receptors (NMDAR) that are critical for excitatory neurotransmission, memory formation, learning, and synaptic plasticity, and metabotropic glutamate receptors (mGluRs) to regulate and fine-tune synaptic signaling over a slower time scale (Niswender and Conn, 2010; Zanetti et al., 2021; Jewett and Thapa, 2026). However, excess glutamate must be rapidly removed from the synaptic cleft because it overstimulates glutamate receptors, causing excessive calcium influx that results in excitotoxic neuronal injury (Lewerenz and Maher, 2015). In fact, chronic glutamate toxicity has been implicated in playing a role in several neurodegenerative diseases, such as Alzheimer’s disease (Lewerenz and Maher, 2015). Astrocytes are responsible for removing excess glutamate via specific excitatory amino acid transporters (EAAT1 and EAAT2) to protect surrounding neurons (Mahmoud et al., 2019). Specifically, the PFC relies on tightly regulated glutamatergic neurotransmission to support executive functions including working memory, cognitive flexibility, attention, inhibitory control, and decision-making (Friedman and Robbins, 2022). Persistent HIV infection within the CNS disrupts glutamate homeostasis through multiple mechanisms elicited by viral proteins (discussed below) and involves infected and activated microglia and reactive astrocytes. As discussed, Tat already causes neurons to be at a higher risk of excitotoxicity due to the loss of fractalkine microglial regulation. Thus, the additional glutamatergic dysregulation of excitatory neurotransmission has far-reaching effects on cortical network function.

**Figure 4:**
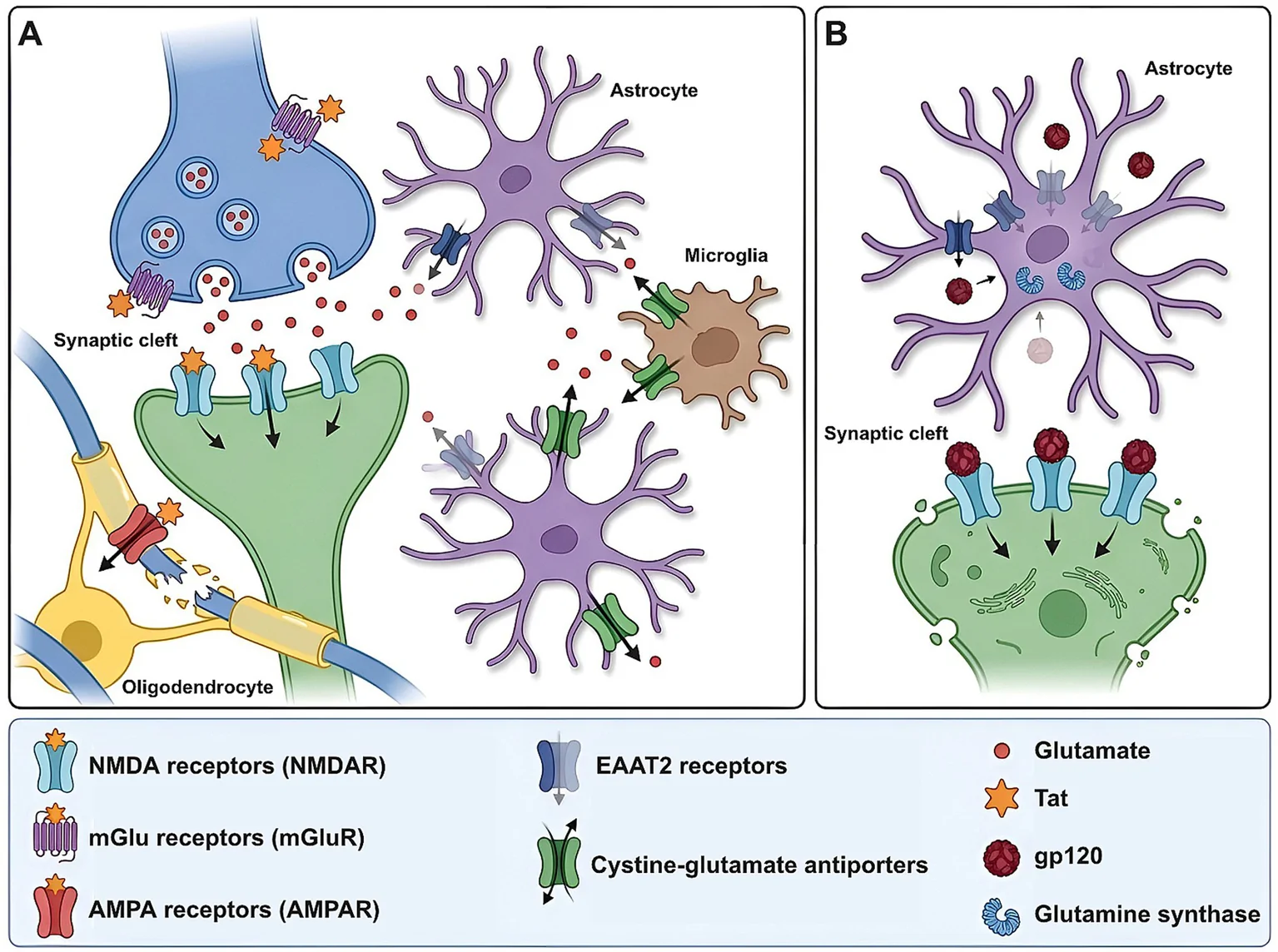
Summary of glutamatergic dysregulation caused by HIV-1 proteins. HIV proteins impair glutamate homeostasis through direct effects on neurons and glial cells, resulting in chronic excitotoxicity and neuronal injury. **(A)** Tat stimulates excessive glutamate release from presynaptic terminals and activated astrocytes and microglia while simultaneously reducing astrocytic glutamate uptake through downregulation of excitatory amino acid transporter 2 (EAAT2). Tat also enhances activation and expression of ionotropic glutamate receptors, including N-methyl-D-aspartate receptors (NMDAR) and *α*-amino-3-hydroxy-5-methyl-4-isoxazolepropionic acid receptors (AMPAR), as well as metabotropic glutamate receptors (mGluRs), resulting in excessive calcium influx, oxidative stress, and excitotoxic neuronal injury. In parallel, inhibition of the astrocytic cystine–glutamate antiporter disrupts glutamate export–import balance and antioxidant homeostasis, further amplifying extracellular glutamate accumulation in synaptic clefts and neurotoxicity. Excitotoxic signaling also contributes to oligodendrocyte dysfunction and myelin damage. **(B)** Gp120 exacerbates glutamatergic dysregulation by suppressing astrocytic glutamine synthetase expression and activity, impairing conversion of glutamate to glutamine and disrupting the glutamate–glutamine cycle. It is also responsible for downregulation of EAAT2 expression in astrocytes. Reduced glutamine recycling, together with diminished glutamate clearance and sustained receptor activation, promotes persistent excitotoxic signaling, synaptic dysfunction, and progressive neuronal degeneration.

Tat has been extensively studied to determines its effects on glutamate dysregulation, and as a result, several mechanisms have been identified (Figure 4A). For an extensive review, please see Duffy et al. (2026). Tat expressed by microglia and astrocytes has been shown to cause direct neurotoxicity by binding to NMDAR and triggering calcium influx (Self et al., 2004). These neurons become highly sensitized to glutamate which triggers a cascade of damage, including neuronal depolarization, mitochondrial instability, and the loss of presynaptic terminals (Duffy et al., 2026). Tat also interacts with AMPARs on oligodendrocytes that causes calcium influx, cell death, and myelin sheath disruption (Zou et al., 2015). Tat can also bind to mGluRs which further increases neuronal excitability and stimulates pathological exocytosis of additional glutamate from nerve endings (Musante et al., 2010; New et al., 1998). This chronic excitatory imbalance results in synaptic dysfunction and is a strong contributor to cognitive impairments seen in HAND. To further exacerbate glutamatergic dysfunction, Tat severely interferes with glutamate clearing by astrocytes. Cell exposure to Tat shows direct downregulation of EAAT2 expression and therefore function (Duffy et al., 2026; Ye et al., 2017). Simultaneously, Tat promotes the upregulation of the cystine-glutamate antiporter in microglia and astrocytes, leading to even more glutamate release into the extracellular space (Marino et al., 2020; Gupta et al., 2010). Together, the toxic buildup of glutamate overwhelms the already diminished clearing capacity of astrocytes as well as overactive synaptic NMDARs, creating a neurotoxic environment characterized by protracted neuroinflammation and excitotoxicity.

Gp120 has also been implicated in glutamate dysregulation, through similar mechanisms to Tat. Human astrocytes exposed to HIV-1 or purified gp120 showed reduced expression of EAAT2 (Figure 4B; Wang et al., 2003). The HIV envelope protein has been shown to reduce the expression of glutamine synthase in astrocytes, which is responsible for converting glutamate into glutamine, effectively neutralizing and completing the clearance role (Chen et al., 2023). Together, gp120 increases the extracellular pool of glutamate around already oversensitive neurons. To further add to the pathology, gp120 has been shown to directly interact with NMDARs, causing neuronal injury in a similar mechanism to Tat (Gemignani et al., 2000; Liu et al., 2023).

Collectively, disruption of glutamatergic signaling compromises PFC circuit integrity, producing deficits in executive function that are characteristic of HAND, and discussed in a subsequent section. Impaired synaptic plasticity and altered neuronal excitability contribute to deficits in attention, cognitive flexibility, planning, inhibitory control, and complex decision-making, even in individuals receiving effective cART. This compounds the neurotoxic effects of HIV-1 proteins already discussed, yet may represent a potential therapeutic target for potentially treating HAND in PLWH (Potter et al., 2013).

### 3.8 Blood–brain barrier disruption

Several studies have elucidated that gp120, Tat, and Nef have direct roles in the disruption of brain microvascular endothelial cells (BMECs) during HIV infection (Table 1). Not only could this provide an entrance mechanism into the CNS for peripherally circulating virus and result in continued seeding of the brain, but it could also cause an increase in proinflammatory cell infiltration even when viral production is undetectable. While HIV is traditionally associated with the infection of immune cells expressing CD4 receptor, human BMECs can be infected (Bobardt et al., 2004). Therefore, many studies have investigated how gp120 binding may affect BBB integrity. A 1998 study using rat BMECs exposed to purified gp120 determined that the BBB increased in permeability to albumin after 24 h (Annunziata et al., 1998). This effect was blocked when cell cultures were incubated with neutralizing gp120 antibodies. An *in vivo* study determined that upon exposing the serum of mice to gp120, there was a significant increase in BBB permeability (Cioni and Annunziata, 2002). To further elucidate the molecular pathways triggered by gp120 to alter BBB integrity, *in vitro* experiments determined that when human BMECs were exposed to gp120 expressed by macrophages and lymphocytes, BBB permeability increased, tightness decreased, and there was an increase in monocyte migration across the barrier (Kanmogne et al., 2007). These effects were blocked when CCR5 antibodies were added, suggesting an essential step of gp120 binding to BMECs to initiate the process. Gp120 can also cause cytoskeletal alterations, morphological changes in BMECs, and increased stress fiber formation (Kanmogne et al., 2007; Shiu et al., 2007). Together, this suggests that gp120 causes BMEC cell retraction by interfering with normal cell structure, leading to physical barrier disruption. Another study by Louboutin and Strayer (2012) determined that purified gp120 injected directly into the caudate putamen of rats caused leakage of vascular contents into the brain, suggesting reduction of BBB integrity. Experiments in this study showed that gp120 was toxic to BMECs, by causing an increase of ROS and lipid peroxidation, which increased the expression of matrix metalloproteinases (MMPs) in the brain after gp120 injection. This led to MMP-mediated destruction of multiple tight junction proteins: laminin, occludin, and claudin-5, explaining the increase in BBB permeability.

The possible role of Tat and BBB integrity was initially studied *in vitro* in cultured BMECs that were exposed to purified Tat for 24 h (András et al., 2003). Here, researchers determined that there was a significant reduction in the expression of several tight junction proteins including claudin-1, claudin-5, and Zonula Occludens 2 (ZO2). *In vivo* analysis of how Tat may effect BBB integrity revealed that in Tat-transgenic mice, Tat expression allowed molecules up to 70 kDa to leak into the brain (Leibrand et al., 2017). Further, after 5 days of Tat induction, fluorescent markers were bilaterally infused into the lateral ventricles. Finally, this study also found that the number of perivascular macrophages and microglia increased in the dorsal striatum of mice expressing Tat. On a molecular level, several pathways have been implicated in how Tat decreases expression of integral tight junction proteins. For a review, see McRae (2016).

Raymond et al. (2016) studied the effects of EV-Nef released from Nef-transfected microglia on the BBB. Here, they determined that EV-Nef alone could disrupt the apical side of the BBB, by reducing transendothelial electrical resistance (TEER) and therefore increasing permeability. Further analysis revealed that Nef triggered a significant decrease in the expression of ZO1, an integral tight junction protein. While the role of EV-Nef in BBB disruption *in vivo* has yet to be elucidated, the study by Raymond et al. (2016) highlights an important contributing factor to the breakdown of BBB integrity during HIV infection.

Together, gp120, Tat, and Nef all work to disrupt BBB permeability by disrupting tight junction proteins, enhance peripheral immune cell migration into the brain, and further contribute to neurotoxicity in the realm of neruoHIV infection. These results highlight how “bystander cells,” both immune and endothelial, are affected by HIV proteins produced during infection, even when viral suppression has been established.

### 3.9 Direct neurotoxicity and CNS cellular injury

Soluble Vpr from infected microglia/macrophages are taken up by neuron precursors, where it binds the inner mitochondrial membrane. This results in increased membrane permeability, an increase in ROS, a reduction of ATP production, and disruption of normal mitochondrial transport within the cytoplasm (Kitayama et al., 2008). Due to mitochondrial dysfunction, inhibition of axonal outgrowth and possible axonal contractions occurs, which leads to synaptic connection failures. The lack of regeneration in an adult brain causes permanent deficits and even accelerates neuronal aging (Wang et al., 2017). Also, there is evidence that Vpr arrests neuron cell cycle at the G2/M phase, which disrupts normal function and leads to neuronal apoptosis (Andersen et al., 2006). Further, Vpr can directly activate caspase-8, once again causing apoptosis (Patel et al., 2000). Therefore, when Vpr is present in neurons, it is responsible for either the disruption or inhibition of axonal connections, or cell death. While not a focus of this review, identical apoptotic processes are experienced by T-cells in response to Vpr, even when not infected with HIV. This suggests a critical role of Vpr in the CD4^+^ T-cell depletion which is a hallmark of HIV/AIDS (Andersen et al., 2006).

In a 2024 study, EV-Nef injected directly into mice brain tissue was shown to impair myelin by directly damaging oligodendrocytes (Schenck et al., 2024). Here, there was a marked decrease in mature oligodendrocytes around the area of EV-Nef injection. This correlated with a loss of myelinated axons. The debris formed by the breakdown of myelin is subsequently cleared by microglia, creating a persistent inflammatory state. *Ex vivo* cerebellar slices were also treated with EV-Nef in this study, which confirmed the *in vivo* results without the influence of systemic immune cells. This confirmed a direct link between Nef and myelin loss. Together, oligodendrocytes lost their complex structures, myelin sheaths could no longer be created or maintained, severe axonal degeneration occurred, and white matter damage was observed.

### 3.10 Summary of HIV protein-mediated pathophysiology on the brain

Several HIV proteins, including gp41, gp120, Nef, Tat, Vpr, and Vpu, orchestrate a multifaceted attack on the brain by triggering chronic proinflammatory responses in microglia through several signaling cascades (Table 1). These proteins drive the secretion of neurotoxic cytokines and increase oxidative stress, often by priming and activating the NLRP3 inflammasome to induce inflammatory cell death via pyroptosis (Figure 2). Specific mediators such as Tat and gp120 further exacerbate damage by initiating ferroptosis (Figure 3). Furthermore, Tat-induced microglial senescence and the dysregulation of the complement system led to excessive pruning of neuronal synapses, directly contributing to the cognitive impairments associated with HAND. Tat can disrupt the CX3CL1-CX3CR1 signaling axis, leading to a reduced neuron-mediated dampening of inflammation, triggering microglial activation. Tat and gp120 have similar roles in interfering with glutamate homeostasis within the CNS, by increasing the sensitivity of neuron receptors to glutamate, decreasing astrocyte-mediated glutamate clearing, as well as causing neurons, microglia and astrocytes to secrete more glutamate (Figure 4). The BBB is also significantly compromised as gp120, Nef, and Tat disrupt integral tight junction proteins, which enhances peripheral immune cell migration and facilitates viral seeding. Direct neurotoxicity occurs as Vpr impairs mitochondrial function and axonal transport in neurons, while Nef damages oligodendrocytes, resulting in myelin loss and white matter injury. These collective mechanisms demonstrate how “bystander” cells are impacted and neuroinflammation persists even when viral suppression is achieved through cART. The synergy between microglial activation, barrier breakdown, and direct neuronal damage establishes a chronic state of inflammation that underscores the complexity of HIV-related neurodegeneration.

## 4 HAND neuroimaging findings

As discussed, HIV proteins can cause synapses to be incorrectly attacked and destroyed, can injure axons, and cause neuronal death. This correlates with well-documented decreased volume of brain structures, such as the caudate nucleus, and associated white matter atrophy (Luo et al., 2025). Brain autopsies of PLWH who developed HAD demonstrated white matter pallor, perivascular infiltrates, and microglial nodules (Ances and Clifford, 2008). As expected, this extreme loss of neuronal communication and brain volume directly correlates with functional impairment and specific neurodegeneration of HAND, which is discussed in the next section.

Due to the HIV-mediated pathophysiological inflammatory effects on the brain, it is not surprising that neuroimaging reveals changes as HAND develops and progresses. MRI imaging is widely used, as it can measure changes in gray and white matter (Luo et al., 2025). Even in patients on cART with viral suppression, changes in brain structure and function can be visualized. A marked reduction in gray matter volume has been detected in multiple regions, especially prefrontal and frontal cortexes, even early in infection (Li et al., 2014). There is a generalized thinning of the cerebral cortex which leads to cognitive dysfunction; however, if cART is initiated soon after infection, this specific finding may be tempered in some PLWH (Sanford et al., 2018). Subcortical atrophy has been detected especially in the caudate nucleus and putamen, which are essential for communication with the PFC, further impacting high-level cognition, motor function, and reward-driven behavior (Becker et al., 2011). White matter is unsurprisingly affected, as HIV can directly and indirectly damage or kill neurons (Underwood et al., 2017; Jensen et al., 2019). Loss of fiber integrity, as well as hyperintensities in white matter, have been observed in patients with HAND, with damage first detected in the corpus callosum, which connects the left and right PFC (Wu et al., 2006; Ragin et al., 2015). Damage here leads to, unsurprisingly, cognitive, behavioral, and emotional regulation challenges. Even in virally suppressed patients, structural connectivity deficits in several brain networks have been detected, such as the occipital and subcortical regions of the PFC (Li et al., 2025). This impairs complex motor controls. Further evidence shows that functional connectivity is decreased in the default mode network, salience network, and executive control network (Luo et al., 2025). When the default network is impaired, patients with HAND will show issues with emotional processing, lack self-reflection or introspection, and struggle with appropriate social interaction. Damage to the salience network leads to challenges switching between the “internal” default network, and “external” executive network. Finally, alterations in the executive network leads to problems with planning, decision-making, goal-directed behavior, etc. (Gross and Grossman, 2010).

Unfortunately, many of these functional and structural changes persist in patients on effective cART, underscoring the ongoing neuroinflammation and neurodegeneration that occurs even in the absence of viral production. As HAND severity increases, the neuroimaging pattern shows increased atrophy, marked white matter disease, and metabolic abnormalities (Haziot et al., 2015). While imaging has helped highlight the specific locations of HIV-driven damage to the brain, no single imaging biomarker is diagnostic. Neuroimaging remains adjunctive to neuropsychological testing to identify specific neurodegenerative signs and symptoms.

## 5 HIV-1 effects on the PFC networks and neuropsychiatric sequelae

The core importance of elucidating the molecular pathophysiology of how HIV infection leads to neurodegeneration is to understand how the clinical presentations of the various forms of HAND present in PLWH, as well as provide potential future therapeutics to counter CNS dysfunction. For example, studies on mice have shown that when Tat is expressed, the PFC is affected and mice experienced recognition memory deficits (Yadav-Samudrala et al., 2025). As discussed, HAND is a tiered disorder, ranging from asymptomatic impairment to dementia (Figure 1). While cART has significantly reduced the number of PLWH who progress to HAD, 50% of patients will develop at least the ANI form of disease. HAND is often a progressive disease, with ANI-diagnosed patients up to six times more likely to progress to symptomatic HAND (Grant et al., 2014).

Neurodegeneration associated with HIV is primarily diagnosed using neuropsychological testing, which requires access to specialized doctors for accurate examination and interpretation of results. For a recent review on obstacles to diagnosing early HAND, see Vastag et al. (2022). It should be noted that several factors not specific to the pathophysiological effects of HIV on the brain can increase the risk of developing HAND (Diaz, 2025). For example, opioid use in PLWH have been linked to higher deficits in cognitive function (Tamargo et al., 2021). Also, low socioeconomic status may be an independent risk factors for HAND development (Diaz, 2025). Regardless, neurocognitive screening, and various biomarkers such as functional MRI or CSF markers for neuroinflammation can help determine if a PLWH has developed HAND.

The earliest indication for HAND is impairment of concentration and attention, working memory issues, and executive dysfunction (Figure 1; Mitra and Sharman, 2026). ANI can often be clinically inapparent, increasing diagnosis difficulty (Mitra and Sharman, 2026). Once symptoms being, it seems that the damage caused by neuroHIV has a significant effect on the PFC and can cause dopaminergic dysfunction. Since the PFC receives input from multiple cortical regions of the brain to process “in the moment” information, it is not surprising that higher order executive functions and working memory are affected, not just “classic” cortical dementia seen in other neurological disorders. The following represents common symptoms of HAND based on synaptodendritic injury and PFC dysfunction: 1. impulse or emotional control: difficulty regulating emotion, speaking before thinking/blurting out inappropriate words; 2. regulating attention: hyperfocusing on one thing, or being easily distracted, difficulty reading; 3. difficulties planning or executing tasks: procrastinating, challenges starting or completing a task; 4. organizational or memory issues: losing track of time, misplacing items; 5. problems with cognitive flexibility: getting stuck on a task, not adapting to changes; 6. behavioral changes: apathy (one of the most common symptoms of HAND), reduced initiative, and emotional blunting (Figure 1) (Mitra and Sharman, 2026; Irollo et al., 2021; Singer and Thames, 2016). Apathy and impaired motivation have been linked to a disruption in dopamine pathways, which links PFC to the basal ganglia (Levy and Czernecki, 2006). It is well-established that HIV neuropathogenesis causes a disrupted dopamine system, primarily through HIV-protein-mediated neuronal damage, and the ability of dopamine to reactivate dormant virus in microglia. This feedback loop further increases neuroinflammation (Basova et al., 2026; Gaskill et al., 2017). As HAND progresses to HAD, psychomotor retardation and subclinical motor signs, such as Parkinson’s-like tremors, can occur (Mitra and Sharman, 2026). This is due to the chronic immune activation and neurotoxic properties of HIV proteins discussed earlier. Depressive symptoms and irritability are more likely to develop, and global dementia appears (Singer and Thames, 2016).

HIV-induced glutamatergic dysregulation, excessive NMDA receptor activation, and dendritic spine loss weaken excitatory circuits of the PFC (Duffy et al., 2026). Chronic neuroinflammation and glutamate-mediated excitotoxicity reduce the signal-to-noise ratio of cortical neuronal activity, impairing sustained attention and increasing distractibility (Cheng et al., 2017). Consequently, individuals with HAND frequently exhibit slower information processing, difficulty concentrating, and impaired performance on attention-demanding tasks (Vastag et al., 2022). Inhibitory control requires coordinated interactions between the PFC, anterior cingulate cortex, and basal ganglia to suppress inappropriate or impulsive responses (Munakata et al., 2011). Excessive glutamatergic signaling alters neuronal excitability and disrupts communication within these circuits, diminishing the ability to inhibit prepotent behaviors. These impairments may contribute to increased impulsivity, reduced behavioral regulation, and difficulty suppressing automatic responses during cognitively demanding situations (Yates, 2024). Cognitive flexibility depends on dynamic modulation of glutamatergic synapses within the PFC and its reciprocal connections with the anterior cingulate cortex and striatum (Medalla and Barbas, 2009; Timothy et al., 2021). HIV-associated disruption in neuronal health, as well as altered NMDA and AMPA receptor trafficking, results in perseverative behavior and reduced ability to shift between tasks or adapt to new rules (Vastag et al., 2022; Mitra and Sharman, 2026). Finally, decision-making relies on a highly interconnected fronto-limbic network (Mesbah et al., 2023; Stoll and Rudebeck, 2024). Glutamatergic projections coordinate communication between these regions to evaluate expected outcomes, assess risk, inhibit maladaptive choices, and guide goal-directed behavior. HIV-mediated disruption of glutamatergic transmission compromises this network integration by, as discussed, weakening functional connectivity between cortical and subcortical circuits. As a result, individuals with HAND may demonstrate impaired judgment, slower decision-making, reduced planning ability, increased susceptibility to risky choices, and difficulty adapting decisions based on feedback (Vastag et al., 2022). Collectively, these circuit-level alterations suggest that glutamatergic dysfunction is not simply a consequence of chronic neuroinflammation but represents a central mechanism linking HIV-induced dysregulation of microglia and astrocytes to impaired synaptic plasticity, disrupted cortical network activity, and the executive dysfunction characteristic of HAND. Persistent abnormalities in glutamate homeostasis may therefore explain why deficits in executive function often remain evident despite effective cART and sustained viral suppression.

There is ample evidence that PLWH experience a high prevalence of neuropsychiatric disorders, such as major depression, bipolar disorder (including AIDS mania), schizophrenia, and substance use disorder (UpToDate, 2026). These disorders have many risk factors, including preexisting psychiatric conditions, affective disorders, and addictions. However, there is a subset of patients that develop new neuropsychiatric sequelae that can only be explained by direct neurological damage and subsequent chronic inflammation of the PFC, dopamine dysregulation, as well as the toxicity of certain antiretroviral drugs (Yuan and Kaul, 2021). Psychiatric care is essential for these patients, especially since PLWH that develop psychiatric disorders also experience higher rates of treatment adherence failure, putting them at greater risk of progressing to AIDS and developing life-threatening opportunistic infections, neoplasms, and increasing their chances of progressing to HAD.

## 6 Potential therapeutic targets to prevent or halt progression of HAND

Since approximately 50% of PLWH will develop at least the mild form of HAND, it is imperative for novel therapeutics to be discovered that could either prevent HAND entirely, or slow/halt progression to the more severe forms of disease. Even mild disease has an impact of the day-to-day lives of PLWH and their support systems. Based on the current knowledge of the pathophysiology of HIV and specific neurological events that lead to structural and functional changes discussed in this review, several avenues could represent potential treatment strategies. However, due to the multifactorial pathogenesis of HIV and HAND (persistent viral reservoirs, activation of multiple neuroinflammatory pathways, permanent synaptodendritic damage etc.) combination or pathway-based regimens will most likely be required, instead of viral-centric treatments alone. Here, several molecular mechanisms and pathways are discussed that may represent potential therapeutic targets to prevent or reduce the severity of HAND.

1. Viral transcription and translation. Since cART fails to eliminate production of early viral proteins such as Tat and Nef that have several roles in neuroHIV pathogenesis, developing drugs that specifically target these processes in cells containing provirus in the CNS would greatly reduce the presence of these neurotoxic proteins, and their downstream effects such as reduced microglial activation or preservation of BBB integrity.

2. The NLRP3 inflammasome and pyroptosis. Multiple HIV proteins can prime and activate the inflammasome, which leads to microglial cell death and release of proinflammatory cytokines. Therapeutics that could prevent the activation of the inflammasome, or a caspase-1 inhibitor that stops production of active IL-1β and IL-18, could be beneficial. Together, these targets could mitigate chronic neuroinflammation and subsequent neuronal damage and loss.

3. Ferroptosis pathways. This self-amplifying neuroinflammatory loop, known to be triggered by Tat and gp120, could be targeted in several ways. Since Tat is known to specifically activate ACSL4, the regulator of ferroptosis activation, a pharmacologic that could block Tat binding may prevent pathway activation, even if intracellular free iron, lipids susceptible to peroxidation, and ROS increase still occur. Alternatively, restoring antioxidant pathways that become dysfunctional during infection could help reduce lipid peroxidation and perhaps prevent subsequent cell membrane perforation.

4. Excessive synaptic pruning. During neuroHIV, C1q and C3 complement pathways become overactivated, which signal already overzealous microglia to inappropriately destroy neuronal synapses. Interventions within the brain could include inhibiting excessive and unwarranted C3 deposition on synapses, or even specifically target astrocyte activity to reduce or eliminate secretion of C3 in response to HIV-induced inflammation.

5. Microglial senescence. Tat causes premature aging of microglia, by increasing expression of cell-cycle arrest proteins p16 and p21 and causing mitochondrial oxidative stress. If a drug was to be designed to bind to and inhibit Tat, this effect on microglia [and many other Tat-mediated pathways (Table 1)], could be alleviated. In fact, a Tat-inhibitor would not only reduce several neuroinflammatory pathways and decrease neuronal damage, but could also reduce or eliminate viral transcription. Without functional Tat, viral RNA transcription would be severely impaired, helping to control viral suppression, and reduce HIV protein production in the CNS.

6. Dopaminergic restoration. Clinically, many HAND symptoms, such as apathy and impaired motivation, are linked to the disruption of dopamine pathways connecting the PFC to the basal ganglia. If therapeutics could restore dopaminergic tone, or protect these specific circuits, they could alleviate some neuropsychiatric sequelae.

7. Glutamatergic homeostasis restoration. The accumulation of excess glutamate due to astrocyte dysfunction, as well as HIV-mediated increase in the expression of excitatory receptors, results in neurotoxicity. Studies have looked to restore normal glutamate homeostasis as a target to treat HAND (Chen et al., 2024). Early work used antagonists against NMDARs, reducing the number of functional receptors on neurons (Raybuck et al., 2017). While *in vitro* and *in vivo* experiments showed neuroprotection upon exposure to NMDAR antagonists, successful clinical trials have been yet to be published. Work has recently been performed to test whether enhancing glutamate uptake in astrocytes could help reduce neurotoxicity through restoring the clearing abilities of these cells, however the long-term efficacy of this remains to be determined (Chen et al., 2024).

8. BBB preservation. Although HIV enters the CNS early in infection, chronic neuroHIV leads to loss of BBB integrity and subsequent increase of BBB permeability. This allows for persistent peripheral virus and immune cell penetration into the brain, enhancing the chronic proinflammatory state and increasing the chance of HAND development or progression to HAD. Since MMPs have been specifically implicated in destruction of tight junction proteins after being activated by HIV proteins, if their activity could be blocked or highly regulated, BBB integrity could be maintained. Also, since gp120 binding to co-receptors located on BMECs, co-receptor antagonists, which already exist as part of cART, may have a role in reducing gp120-mediated barrier disruption. Several studies have investigated different classes of inhibitors to prevent gp120-mediated BBB compromise, for example cannabinoid agonists, which prevented downregulation of BBB TJ proteins (Lu et al., 2008). However, studies will need to continue to see their effectiveness prospectively on HAND in *in vivo* studies, potentially analyzing their effects prospectively in PLWH.

9. Direct inhibition of HIV proteins. As mentioned, inhibiting Tat directly, via an antagonist or neutralizing antibody, may reduce, or block entirely, the downstream effects of the protein on CNS and PFC integrity. One could argue that any other HIV protein discussed in this review could be a potential target for antagonistic treatments that cross the BBB, especially if coupled with viral transcription inhibitors to further reduce the pool of HIV proteins and viral production. This will require understanding of the direct molecular mechanisms and amino acid-level interactions between HIV proteins and their cellular targets.

## 7 Conclusion

Despite the significant successes of cART in reducing HIV-associated morbidity and mortality, HAND remains a persistent clinical challenge. This spectrum of disorders ranges from asymptomatic neurocognitive impairment, with subtle deficits in concentration, executive function, and memory that does not significantly impact daily living, to severe dementia. A critical limitation of current cART regimens is their inability to halt the transcription and translation of the HIV provirus, which is established very early in the CNS in resident microglia. Consequently, neurotoxic viral proteins continue to be produced and secreted even in patients with undetectable peripheral viral loads. This establishes a state of chronic, damaging neuroinflammation and synaptic dysfunction.

The pathophysiology of HAND is primarily orchestrated by the sustained activation of microglia and downstream effects. These cells facilitate neurodegeneration through several overlapping mechanisms: the priming and activation of the inflammasome, the induction of ferroptosis, and the promotion of microglial senescence. Furthermore, HIV-induced inflammation drives astrocytes to secrete complement factors which incorrectly tag healthy synapses for pruning, as well as interfering with the glutamate clearing responsibilities. Finally, the simultaneous disruption of the BBB allows for continued peripheral immune cell infiltration and subsequent enhancement of an already-established proinflammatory state. These collective molecular insults result over time in the loss of CNS fiber integrity.

The structural and functional changes seen in HAND (especially HAD), particularly within the PFC and its associated subcortical networks, culminate in significant deficits. Neuroimaging frequently reveals gray matter thinning and decreased connectivity in networks essential for executive control and emotional regulation. Clinically, these disruptions manifest as impairment in working memory, attention and cognitive flexibility, and are often accompanied by neuropsychiatric sequelae such as apathy, bipolar disorder, and schizophrenia. The persistence of neurotoxic markers, such as Tat, despite effective viral suppression, underscores the urgent need for novel therapeutic strategies that target both viral protein synthesis and specific inflammatory and neurotransmitter pathways to prevent or mitigate the long-term impact of HAND. It should be cautioned that PFC damage caused by HIV can occur early in infection, perhaps even before a patient is diagnosed. The timing and implementation of therapeutics relating to HAND will most likely require a multi-disciplinary approach and individualized to the needs of each patient.

## References

[ref1] Ances B. M. Clifford D. B. (2008). HIV-associated neurocognitive disorders and the impact of combination antiretroviral therapies. Curr. Neurol. Neurosci. Rep. 8, 455–461. doi: 10.1007/s11910-008-0073-3 18957181 PMC3932327

[ref2] Andersen J. L. DeHart J. L. Zimmerman E. S. Ardon O. Kim B. Jacquot G. . (2006). HIV-1 Vpr-induced apoptosis is cell cycle dependent and requires Bax but not ANT. PLoS Pathog. 2:e127. doi: 10.1371/journal.ppat.0020127, 17140287 PMC1665652

[ref3] András I. E. Pu H. Deli M. A. Nath A. Hennig B. Toborek M. (2003). HIV-1 tat protein alters tight junction protein expression and distribution in cultured brain endothelial cells. J. Neurosci. Res. 74, 255–265. doi: 10.1002/jnr.10762 14515355

[ref4] Annunziata P. Cioni C. Toneatto S. Paccagnini E. (1998). HIV-1 gp120 increases the permeability of rat brain endothelium cultures by a mechanism involving substance P. AIDS 12, 2377–2385. doi: 10.1097/00002030-199818000-00006 9875575

[ref5] Bachstetter A. D. Morganti J. M. Jernberg J. Schlunk A. Mitchell S. H. Brewster K. W. . (2011). Fractalkine and CX3CR1 regulate hippocampal neurogenesis in adult and aged rats. Neurobiol. Aging 32, 2030–2044. doi: 10.1016/j.neurobiolaging.2009.11.022 20018408 PMC2889032

[ref6] Basova L. V. Riley T. Delorme-Walker V. de Siqueira-Lima D. Milner R. Ellis R. J. . (2026). Distinct roles of dopamine receptors in HIV latency reversal in a myeloid cell model. Front. Immunol. 17:1817754. doi: 10.3389/fimmu.2026.1817754 42292415 PMC13253437

[ref7] Battistini Garcia S. A. Zubair M. Guzman N. (2026). “CD4 cell count and HIV,” in StatPearls, (Treasure Island, FL: StatPearls Publishing). 30020661

[ref8] Becker J. T. Sanders J. Madsen S. K. Ragin A. Kingsley L. Maruca V. . (2011). Subcortical brain atrophy persists even in HAART-regulated HIV disease. Brain Imaging Behav. 5, 77–85. doi: 10.1007/s11682-011-9113-8, 21264551 PMC3082694

[ref9] Ben Haij N. Leghmari K. Planès R. Thieblemont N. Bahraoui E. (2013). HIV-1 Tat protein binds to TLR4-MD2 and signals to induce TNF-α and IL-10. Retrovirology 10:123. doi: 10.1186/1742-4690-10-123 24165011 PMC4231456

[ref10] Bento D. Nguyen A. D. (2026). “HIV-1–associated opportunistic infections,” in StatPearls [Internet], (Treasure Island, FL: StatPearls Publishing).

[ref11] Bobardt M. D. Salmon P. Wang L. Esko J. D. Gabuzda D. Fiala M. . (2004). Contribution of proteoglycans to human immunodeficiency virus type 1 brain invasion. J. Virol. 78, 6567–6584. doi: 10.1128/JVI.78.12.6567-6584.2004 15163749 PMC416544

[ref12] Borrajo A. Spuch C. Penedo M. A. Olivares J. M. Agís-Balboa R. C. (2021). Important role of microglia in HIV-1 associated neurocognitive disorders and the molecular pathways implicated in its pathogenesis. Ann. Med. 53, 43–69. doi: 10.1080/07853890.2020.1814962 32841065 PMC7877929

[ref13] Cardona A. E. Pioro E. P. Sasse M. E. Kostenko V. Cardona S. M. Dijkstra I. M. . (2006). Control of microglial neurotoxicity by the fractalkine receptor. Nat. Neurosci. 9, 917–924. doi: 10.1038/nn1715 16732273

[ref14] Centers for Disease Control (CDC) (1981). Kaposi’s sarcoma and *pneumocystis* pneumonia among homosexual men--New York City and California. MMWR Morb. Mortal Wkly. Rep. 30, 305–308, 6789108

[ref15] Chafee M. V. Heilbronner S. R. (2022). Prefrontal cortex. Curr. Biol. 32, R346–R351. doi: 10.1016/j.cub.2022.02.071 35472417, 35472417 PMC9832551

[ref16] Chen J. Zou J. Gao X. Cao H. (2023). HIV-1 gp120 induces glutamate excitotoxicity by diminishing glutamine Synthetase expression in astrocytes. Blood. 142: 5365. doi:10.1182/blood-2023-184411.

[ref17] Chen J. Zou J. Huang P. Gao X. Lun J. Li Y. . (2024). KYNA ameliorates glutamate toxicity of HAND by enhancing glutamate uptake in A2 astrocytes. Int. J. Mol. Sci. 25:4286. doi: 10.3390/ijms25084286 38673879 PMC11050540

[ref18] Cheng Y. Jung J. Guo L. Shuboni-Mulligan D. D. Chen J. F. Hu W. . (2025). HIV-TAT dysregulates microglial lipid metabolism through SREBP2/miR-124 axis: implication of lipid droplet accumulation microglia in NeuroHIV. Brain Behav. Immun. 123, 108–122. doi: 10.1016/j.bbi.2024.09.011 39260763 PMC11624073

[ref19] Cheng J. Liu A. Shi M. Y. Yan Z. (2017). Disrupted glutamatergic transmission in prefrontal cortex contributes to behavioral abnormality in an animal model of ADHD. Neuropsychopharmacology 42, 2096–2104. doi: 10.1038/npp.2017.30 28176786 PMC5561342

[ref20] Cioni C. Annunziata P. (2002). Circulating gp120 alters the blood–brain barrier permeability in HIV-1 gp120 transgenic mice. Neurosci. Lett. 330, 299–301. doi: 10.1016/S0304-3940(02)00814-5, 12270651

[ref21] Coffin J. M. Hughes S. H. Varmus H. E. (1997). “Cellular targets of infection,” in Retroviruses, (Cold Spring Harbor (NY): Cold Spring Harbor Laboratory Press). 21433340

[ref22] Davé V. A. Klein R. S. (2023). The multitaskers of the brain: glial responses to viral infections and associated post-infectious neurologic sequelae. Glia 71, 803–818. doi: 10.1002/glia.24294 36334073 PMC9931640

[ref23] de Sousa A. F. Zeidler Z. E. Almeida-Filho D. G. . (2026). The prefrontal cortex controls memory organization in the hippocampus. Nat Neurosci 29, 1191–1202. doi: 10.1038/s41593-026-02231-1 42049880 PMC13156042

[ref24] Deeks S. G. Overbaugh J. Phillips A. Buchbinder S. (2015). HIV infection. Nat. Rev. Dis. Primers 1:15035. doi: 10.1038/nrdp.2015.35, 27188527

[ref25] Deshmane S. L. Mukerjee R. Fan S. Del Valle L. Michiels C. Sweet T. . (2009). Activation of the oxidative stress pathway by HIV-1 Vpr leads to induction of hypoxia-inducible factor 1alpha expression. J. Biol. Chem. 284, 11364–11373. doi: 10.1074/jbc.M809266200 19204000 PMC2670142

[ref26] Diaz M. M. (2025). Update on neurological complications of HIV. Curr. Opin. HIV AIDS 20, 337–343. doi: 10.1097/COH.0000000000000951 40397601 PMC12414629

[ref27] Dixon S. J. Lemberg K. M. Lamprecht M. R. Skouta R. Zaitsev E. M. Gleason C. E. . (2012). Ferroptosis: an iron-dependent form of nonapoptotic cell death. Cell 149, 1060–1072. doi: 10.1016/j.cell.2012.03.042 22632970 PMC3367386

[ref28] Donoso M. D’Amico D. Valdebenito S. Hernandez C. A. Prideaux B. Eugenin E. A. (2022). Identification, quantification, and characterization of HIV-1 reservoirs in the human brain. Cells 11:2379. doi: 10.3390/cells11152379 35954221 PMC9367788

[ref29] Duan M. Yao H. Cai Y. Liao K. Seth P. Buch S. (2014). HIV-1 Tat disrupts CX3CL1-CX3CR1 axis in microglia via the NF-κBYY1 pathway. Curr. HIV Res. 12, 189–200. doi: 10.2174/1570162x12666140526123119 24862326 PMC5003019

[ref30] Duffy B. C. Nonnemacher M. R. Kortagere S. (2026). HIV-1 Tat-driven glutamate dysregulation: implications for cognitive impairment in HAND. Curr. HIV/AIDS Rep. 23:18. doi: 10.1007/s11904-026-00789-w 42295509 PMC13269467

[ref31] Eggers C. Arendt G. Hahn K. Husstedt I. W. Maschke M. Neuen-Jacob E. . (2017). HIV-1-associated neurocognitive disorder: epidemiology, pathogenesis, diagnosis, and treatment. J. Neurol. 264, 1715–1727. doi: 10.1007/s00415-017-8503-2 28567537 PMC5533849

[ref32] Ene L. Duiculescu D. Ruta S. (2011). How much do antiretroviral drugs penetrate into the central nervous system? J. Med. Life 4, 432–439., 22514580 PMC3227164

[ref33] Engelman A. Cherepanov P. (2012). The structural biology of HIV-1: mechanistic and therapeutic insights. Nat. Rev. Microbiol. 10, 279–290. doi: 10.1038/nrmicro2747, 22421880 PMC3588166

[ref34] Everall I. P. Heaton R. K. Marcotte T. D. Ellis R. J. McCutchan J. A. Atkinson J. H. . (1999). Cortical synaptic density is reduced in mild to moderate human immunodeficiency virus neurocognitive disorder. HNRC Group. HIV Neurobehavioral Research Center. Brain Pathol. 9, 209–217. doi: 10.1111/j.1750-3639.1999.tb00219.x, 10219738 PMC8098484

[ref35] Faust T. B. Binning J. M. Gross J. D. Frankel A. D. (2017). Making sense of multifunctional proteins: human immunodeficiency virus type 1 accessory and regulatory proteins and connections to transcription. Annu Rev Virol. 4, 241–260. doi: 10.1146/annurev-virology-101416-041654 28961413 PMC5750048

[ref36] Friedman N. P. Robbins T. W. (2022). The role of prefrontal cortex in cognitive control and executive function. Neuropsychopharmacology 47, 72–89. doi: 10.1038/s41386-021-01132-0 34408280 PMC8617292

[ref37] Gaskill P. J. Miller D. R. Gamble-George J. Yano H. Khoshbouei H. (2017). HIV, tat and dopamine transmission. Neurobiol. Dis. 105, 51–73. doi: 10.1016/j.nbd.2017.04.015, 28457951 PMC5541386

[ref38] Gemignani A. Paudice P. Pittaluga A. Raiteri M. (2000). The HIV-1 coat protein gp120 and some of its fragments potently activate native cerebral NMDA receptors mediating neuropeptide release. Eur. J. Neurosci. 12, 2839–2846. doi: 10.1046/j.1460-9568.2000.00172.x 10971626

[ref39] German Advisory Committee Blood (Arbeitskreis Blut), Subgroup ‘Assessment of Pathogens Transmissible by Blood’ (2016). Human immunodeficiency virus (HIV). Transfus. Med. Hemother. 43, 203–222. doi: 10.1159/000445852 27403093 PMC4924471

[ref40] Gisslén M. Price R. W. Nilsson S. (2011). The definition of HIV-associated neurocognitive disorders: are we overestimating the real prevalence? BMC Infect. Dis. 11:356. doi: 10.1186/1471-2334-11-356 22204557 PMC3260107

[ref41] Gomez-Arboledas A. Acharya M. M. Tenner A. J. (2021). The role of complement in synaptic pruning and neurodegeneration. Immunotargets Ther. 10, 373–386. doi: 10.2147/ITT.S305420 34595138 PMC8478425

[ref42] Gradisnik L. Velnar T. (2023). Astrocytes in the central nervous system and their functions in health and disease: a review. World J. Clin. Cases 11, 3385–3394. doi: 10.12998/wjcc.v11.i15.3385, 37383914 PMC10294192

[ref43] Grant I. Franklin D. R. Deutsch R. Woods S. P. Vaida F. Ellis R. J. . (2014). Asymptomatic HIV-associated neurocognitive impairment increases risk for symptomatic decline. Neurology 82, 2055–2062. doi: 10.1212/WNL.0000000000000492, 24814848 PMC4118496

[ref44] Gross R. G. Grossman M. (2010). Executive resources. Continuum (Minneap Minn). 16, 140–152. doi: 10.1212/01.CON.0000368266.46038.0e, 22810519 PMC3652226

[ref45] Gupta S. Knight A. G. Gupta S. Knapp P. E. Hauser K. F. Keller J. N. . (2010). HIV Tat elicits microglial glutamate release: role of NAPDH oxidase and the cystine-glutamate antiporter. Neurosci. Lett. 485, 233–236. doi: 10.1016/j.neulet.2010.09.019, 20849923 PMC2956797

[ref46] Haij N. B. Planès R. Leghmari K. Serrero M. Delobel P. Izopet J. . (2015). HIV-1 Tat protein induces production of proinflammatory cytokines by human dendritic cells and monocytes/macrophages through engagement of TLR4-MD2-CD14 complex and activation of NF-κB pathway. PLoS One 10:e0129425. doi: 10.1371/journal.pone.0129425 26090662 PMC4474861

[ref47] Halcrow P. W. Lakpa K. L. Khan N. Afghah Z. Miller N. Datta G. . (2022). HIV-1 gp120-induced endolysosome de-acidification leads to efflux of endolysosome iron, and increases in mitochondrial iron and reactive oxygen species. J. Neuroimmune Pharmacol. 17, 181–194. doi: 10.1007/s11481-021-09995-2, 33834418 PMC8497638

[ref48] Harrison J. K. Jiang Y. Chen S. Xia Y. Maciejewski D. McNamara R. K. . (1998). Role for neuronally derived fractalkine in mediating interactions between neurons and CX3CR1-expressing microglia. Proc. Natl. Acad. Sci. USA 95, 10896–10901. doi: 10.1073/pnas.95.18.10896, 9724801 PMC27992

[ref49] Hasan A. R. Tasnim F. Aktaruzzaman M. Islam M. T. Rayhan R. Brishti A. . (2024). The alteration of microglial calcium homeostasis in central nervous system disorders: a comprehensive review. Neuroglia 5, 410–444. doi: 10.3390/neuroglia5040027

[ref50] Hathaway W. R. Newton B. W. (2026). “Neuroanatomy, prefrontal cortex,” in StatPearls, (Treasure Island, FL: StatPearls Publishing). 29763094

[ref51] Haziot M. E. J. Barbosa Junior S. P. Vidal J. E. de Oliveira F. T. M. de Oliveira A. C. P. (2015). Neuroimaging of HIV-associated neurocognitive disorders. Dement. Neuropsychol. 9, 380–384. doi: 10.1590/1980-57642015DN94000380, 29213987 PMC5619320

[ref52] Hazleton J. E. Berman J. W. Eugenin E. A. (2010). Novel mechanisms of central nervous system damage in HIV infection. HIV AIDS 2, 39–49. doi: 10.2147/hiv.s9186 PMC3218694 22096383

[ref53] He X. Yang W. Zeng Z. Wei Y. Gao J. Zhang B. . (2020). NLRP3-dependent pyroptosis is required for HIV-1 gp120-induced neuropathology. Cell. Mol. Immunol. 17, 283–299. doi: 10.1038/s41423-019-0260-y 31320730 PMC7052202

[ref54] Hu S. Ali H. Sheng W. S. Ehrlich L. C. Peterson P. K. Chao C. C. (1999). Gp-41-mediated astrocyte inducible nitric oxide synthase mRNA expression: involvement of interleukin-1β production by microglia. J. Neurosci. 19, 6468–6474. 10414975 10.1523/JNEUROSCI.19-15-06468.1999 PMC6782827

[ref55] Irollo E. Luchetta J. Ho C. Nash B. Meucci O. (2021). Mechanisms of neuronal dysfunction in HIV-associated neurocognitive disorders. Cell. Mol. Life Sci. 78, 4283–4303. doi: 10.1007/s00018-021-03785-y 33585975 PMC8164580

[ref56] Jensen B. K. Roth L. M. Grinspan J. B. Jordan-Sciutto K. L. (2019). White matter loss and oligodendrocyte dysfunction in HIV: a consequence of the infection, the antiretroviral therapy or both? Brain Res. 1724:146397. doi: 10.1016/j.brainres.2019.146397 31442414 PMC6779527

[ref57] Jewett B. E. Thapa B. (2026). “Physiology, NMDA receptor,” in StatPearls, (Treasure Island, FL: StatPearls Publishing). 30137779

[ref58] Jiao L. Li X. Luo Y. Wei J. Ding X. Xiong H. . (2022). Iron metabolism mediates microglia susceptibility in ferroptosis. Front. Cell. Neurosci. 16:995084. doi: 10.3389/fncel.2022.995084 36111246 PMC9469838

[ref59] Joint United Nations Programme on HIV/AIDS (2025). AIDS, Crisis and Power to Transform: UNAIDS Global AIDS Update 2025. Geneva: Joint United Nations Programme on HIV/AIDS.

[ref60] Kanmogne G. D. Schall K. Leibhart J. Knipe B. Gendelman H. E. Persidsky Y. (2007). HIV-1 gp120 compromises blood–brain barrier integrity and enhance monocyte migration across blood–brain barrier: implication for viral neuropathogenesis. J. Cereb. Blood Flow Metab. 27, 123–134. doi: 10.1038/sj.jcbfm.9600330 16685256 PMC2232899

[ref61] Kannan M. Sil S. Oladapo A. Thangaraj A. Periyasamy P. Buch S. (2023). HIV-1 Tat-mediated microglial ferroptosis involves the miR-204-ACSL4 signaling axis. Redox Biol. 62:102689. doi: 10.1016/j.redox.2023.102689 37023693 PMC10106521

[ref62] Kannan M. Singh S. Chemparathy D. T. Oladapo A. A. Gawande D. Y. Dravid S. M. . (2022). HIV-1 Tat induced microglial EVs leads to neuronal synaptodendritic injury: microglia-neuron cross-talk in NeuroHIV. Extracell Vesicles Circ Nucl Acids. 3, 133–149. doi: 10.20517/evcna.2022.14 36812097 PMC9937449

[ref63] Khan N. Halcrow P. W. Lakpa L. K. Rehan M. Chen X. Geiger J. D. (2021). Endolysosome iron restricts Tat-mediated HIV-1 LTR transactivation by increasing HIV-1 Tat oligomerization and β-catenin expression. J. Neurovirol. 27, 755–773. doi: 10.1007/s13365-021-01016-5, 34550543 PMC8602775

[ref64] Kitayama H. Miura Y. Ando Y. Hoshino S. Ishizaka Y. Koyanagi Y. (2008). Human immunodeficiency virus type 1 Vpr inhibits axonal outgrowth through induction of mitochondrial dysfunction. J. Virol. 82, 2528–2542. doi: 10.1128/JVI.02094-07 18094160 PMC2258941

[ref65] Klingler J. Anton H. Réal E. Zeiger M. Moog C. Mély Y. . (2020). How HIV-1 gag manipulates its host cell proteins: a focus on interactors of the Nucleocapsid domain. Viruses 12:888. doi: 10.3390/v12080888 32823718 PMC7471995

[ref66] Kogan M. Rappaport J. (2011). HIV-1 accessory protein Vpr: relevance in the pathogenesis of HIV and potential for therapeutic intervention. Retrovirology 8:25. doi: 10.1186/1742-4690-8-25 21489275 PMC3090340

[ref67] Kwong P. D. Wyatt R. Robinson J. Sweet R. W. Sodroski J. Hendrickson W. A. (1998). Structure of an HIV gp120 envelope glycoprotein in complex with the CD4 receptor and a neutralizing human antibody. Nature 393, 648–659. doi: 10.1038/31405 9641677 PMC5629912

[ref68] Leibrand C. R. Paris J. J. Ghandour M. S. Knapp P. E. Kim W. K. Hauser K. F. . (2017). HIV-1 tat disrupts blood-brain barrier integrity and increases phagocytic perivascular macrophages and microglia in the dorsal striatum of transgenic mice. Neurosci. Lett. 640, 136–143. doi: 10.1016/j.neulet.2016.12.073 28057474 PMC5401762

[ref69] Levy R. Czernecki V. (2006). Apathy and the basal ganglia. J. Neurol. 253, VII54–VII61. doi: 10.1007/s00415-006-7012-5, 17131230

[ref70] Lewerenz J. Maher P. (2015). Chronic glutamate toxicity in neurodegenerative diseases—what is the evidence? Front. Neurosci. 9:469. doi: 10.3389/fnins.2015.00469 26733784 PMC4679930

[ref71] Li J. Cao F. Yin H. l. Huang Z. j. Lin Z. t. Mao N. . (2020). Ferroptosis: past, present and future. Cell Death Dis. 11:88. doi: 10.1038/s41419-020-2298-2, 32015325 PMC6997353

[ref72] Li F. Chen X. (2026). Recent progress in understanding ferroptosis mechanisms in infectious diseases. Front. Cell. Infect. Microbiol. 16:1778721. doi: 10.3389/fcimb.2026.1778721 41982455 PMC13071034

[ref73] Li Z. Jin X. Zhang M. Wang H. Liu W. Wang B. . (2025). Divergent structural and functional brain alterations in HIV-infected patients: a multimodal meta-analysis. Front. Neurol. 16. doi: 10.3389/fneur.2025.1618408, 40901666 PMC12400857

[ref74] Li Y. Li H. Gao Q. Yuan D. Zhao J. (2014, 2014). Structural gray matter change early in male patients with HIV. Int. J. Clin. Exp. Med. 7, 3362–3369. 25419369 PMC4238549

[ref75] Li L. Li H. S. Pauza C. D. Bukrinsky M. Zhao R. Y. (2005). Roles of HIV-1 auxiliary proteins in viral pathogenesis and host-pathogen interactions. Cell Res. 15, 923–934. doi: 10.1038/sj.cr.7290370, 16354571

[ref76] Lichterfeld M. Gao C. Yu X. G. (2022). An ordeal that does not heal: understanding barriers to a cure for HIV-1 infection. Trends Immunol. 43, 608–616. doi: 10.1016/j.it.2022.06.002 35905706 PMC9346997

[ref77] Limatola C. Lauro C. Catalano M. Ciotti M. T. Bertollini C. Di Angelantonio S. . (2005). Chemokine CX3CL1 protects rat hippocampal neurons against glutamate-mediated excitotoxicity. J. Neuroimmunol. 166, 19–28. doi: 10.1016/j.jneuroim.2005.03.023, 16019082

[ref78] Liu J. Xie J. Dutta D. Xiong H. (2023). HIV-1 envelope protein gp120 modulation of glutamate effects on cortical neuronal synapses: implications for HIV-1-associated neuropathogenesis. Int. J. Physiol. Pathophysiol. Pharmacol. 15, 75–87., 37457651 PMC10349318

[ref79] Louboutin J. P. Strayer D. S. (2012). Blood-brain barrier abnormalities caused by HIV-1 gp120: mechanistic and therapeutic implications. ScientificWorldJournal 2012:482575. doi: 10.1100/2012/482575 22448134 PMC3289936

[ref80] Lu T. S. Avraham H. K. Seng S. Tachado S. D. Koziel H. Makriyannis A. . (2008). Cannabinoids inhibit HIV-1 Gp120-mediated insults in brain microvascular endothelial cells. J. Immunol. 181, 6406–6416. doi: 10.4049/jimmunol.181.9.6406 18941231 PMC3735224

[ref81] Lu S. M. Tremblay M. È. King I. L. Qi J. Reynolds H. M. Marker D. F. . (2011). HIV-1 Tat-induced microgliosis and synaptic damage via interactions between peripheral and central myeloid cells. PLoS One 6:e23915. doi: 10.1371/journal.pone.0023915, 21912650 PMC3166280

[ref82] Luckheeram R. V. Zhou R. Verma A. D. Xia B. (2012). CD4+T cells: differentiation and functions. Clin. Dev. Immunol. 2012:925135. doi: 10.1155/2012/925135 22474485, 22474485 PMC3312336

[ref83] Lundquist C. A. Tobiume M. Zhou J. Unutmaz D. Aiken C. (2002). Nef-mediated downregulation of CD4 enhances human immunodeficiency virus type 1 replication in primary T lymphocytes. J. Virol. 76, 4625–4633. doi: 10.1128/JVI.76.9.4625-4633.2002 11932428 PMC155097

[ref84] Luo H. Chen J. Liu J. Wang W. Hou C. Jiang X. . (2025). Bridging brain and blood: a prospective view on neuroimaging-exosome correlations in HIV-associated neurocognitive disorders. Front. Neurol. 15:15. doi: 10.3389/fneur.2024.1479272, 39839878 PMC11745957

[ref85] Mahmoud S. Gharagozloo M. Simard C. Gris D. (2019). Astrocytes maintain glutamate homeostasis in the CNS by controlling the balance between glutamate uptake and release. Cells 8:184. doi: 10.3390/cells8020184 30791579 PMC6406900

[ref86] Maldonado K. A. Alsayouri K. (2026). “Physiology, brain,” in StatPearls, (Treasure Island (FL): StatPearls Publishing). 31869182

[ref87] Malvaso A. Gatti A. Negro G. Calatozzolo C. Medici V. Poloni T. E. (2023). Microglial senescence and activation in healthy aging and Alzheimer’s disease: systematic review and neuropathological scoring. Cells 12:2824. doi: 10.3390/cells12242824 38132144 PMC10742050

[ref88] Mamik M. K. Hui E. Branton W. G. McKenzie B. A. Chisholm J. Cohen E. A. . (2017). HIV-1 viral protein R activates NLRP3 inflammasome in microglia: implications for HIV-1 associated neuroinflammation. J. Neuroimmune Pharmacol. 12, 233–248. doi: 10.1007/s11481-016-9708-3, 27726055

[ref89] Marino J. Wigdahl B. Nonnemacher M. R. (2020). Extracellular HIV-1 Tat mediates increased glutamate in the CNS leading to onset of senescence and progression of HAND. Front. Aging Neurosci. 12:12. doi: 10.3389/fnagi.2020.00168, 32581774 PMC7295946

[ref90] Mason S. J. Sreeram S. Niazi F. Leskov K. Levine A. D. Karn J. . (2025). HIV infection in microglia leads to senescence, triggering activation of neurotoxicity pathways. bioRxiv:2025.05.08.651477. doi: 10.1101/2025.05.08.651477, 40654822 PMC12247681

[ref91] Mattison H. A. Nie H. Gao H. Zhou H. Hong J. S. Zhang J. (2013). Suppressed pro-inflammatory response of microglia in CX3CR1 knockout mice. J. Neuroimmunol. 257, 110–115. doi: 10.1016/j.jneuroim.2013.02.008, 23499256 PMC3702048

[ref92] McGuire J. L. Gill A. J. Douglas S. D. Kolson D. L. (2016). The complement system, neuronal injury, and cognitive function in horizontally-acquired HIV-infected youth. J. Neurovirol. 22, 823–830. doi: 10.1007/s13365-016-0460-5 27273074 PMC5127892

[ref93] McLaurin K. A. Li H. Booze R. M. Mactutus C. F. (2019). Disruption of timing: NeuroHIV progression in the post-cART era. Sci. Rep. 9:827. doi: 10.1038/s41598-018-36822-1, 30696863 PMC6351586

[ref94] McRae M. (2016). HIV and viral protein effects on the blood brain barrier. Tissue Barriers. 4:e1143543. doi: 10.1080/21688370.2016.1143543 27141423 PMC4836474

[ref95] Medalla M. Barbas H. (2009). Synapses with inhibitory neurons differentiate anterior cingulate from dorsolateral prefrontal pathways associated with cognitive control. Neuron 61, 609–620. doi: 10.1016/j.neuron.2009.01.006, 19249280 PMC2804928

[ref96] Mesbah R. Koenders M. A. van der Wee N. J. A. Giltay E. J. van Hemert A. M. de Leeuw M. (2023). Association between the Fronto-limbic network and cognitive and emotional functioning in individuals with bipolar disorder. JAMA Psychiatry 80, 432–440. doi: 10.1001/jamapsychiatry.2023.0131 36988918 PMC10061312

[ref97] Michell-Robinson M. A. Touil H. Healy L. M. Owen D. R. Durafourt B. A. Bar-Or A. . (2015). Roles of microglia in brain development, tissue maintenance and repair. Brain 138, 1138–1159. doi: 10.1093/brain/awv066, 25823474 PMC5963417

[ref98] Mitra P. Sharman T. (2026). “HIV Neurocognitive Disorders,” in StatPearls, (Treasure Island, FL: StatPearls Publishing). 32310414

[ref99] Mohabbat A. Bannazadeh Baghi H. (2025). Chronic neuroplasticity changes following neurotropic viral infection: mechanisms and implications. Cell. Mol. Neurobiol. 45:94. doi: 10.1007/s10571-025-01622-5 41186612 PMC12586820

[ref100] Mukhamedova N. Hoang A. Dragoljevic D. Dubrovsky L. Pushkarsky T. Low H. . (2019). Exosomes containing HIV protein Nef reorganize lipid rafts potentiating inflammatory response in bystander cells. PLoS Pathog. 15:e1007907. doi: 10.1371/journal.ppat.1007907, 31344124 PMC6657916

[ref101] Munakata Y. Herd S. A. Chatham C. H. Depue B. E. Banich M. T. O’Reilly R. C. (2011). A unified framework for inhibitory control. Trends Cogn. Sci. 15, 453–459. doi: 10.1016/j.tics.2011.07.011 21889391 PMC3189388

[ref102] Musante V. Summa M. Neri E. Puliti A. Godowicz T. T. Severi P. . (2010). The HIV-1 viral protein Tat increases glutamate and decreases GABA exocytosis from human and mouse neocortical nerve endings. Cereb. Cortex 20, 1974–1984. doi: 10.1093/cercor/bhp274 20034999

[ref103] New D. R. Maggirwar S. B. Epstein L. G. Dewhurst S. Gelbard H. A. (1998). HIV-1 Tat induces neuronal death via tumor necrosis factor-alpha and activation of non-N-methyl-D-aspartate receptors by a NFkappaB-independent mechanism. J. Biol. Chem. 273, 17852–17858. doi: 10.1074/jbc.273.28.17852 9651389

[ref104] Nightingale S. Ances B. Cinque P. Dravid A. Dreyer A. J. Gisslén M. . (2023). Cognitive impairment in people living with HIV: consensus recommendations for a new approach. Nat. Rev. Neurol. 19, 424–433. doi: 10.1038/s41582-023-00813-2, 37311873

[ref105] Niswender C. M. Conn P. J. (2010). Metabotropic glutamate receptors: physiology, pharmacology, and disease. Annu. Rev. Pharmacol. Toxicol. 50, 295–322. doi: 10.1146/annurev.pharmtox.011008.145533 20055706 PMC2904507

[ref106] Nitkiewicz J. Borjabad A. Morgello S. Murray J. Chao W. Emdad L. . (2017). HIV induces expression of complement component C3 in astrocytes by NF-κB-dependent activation of interleukin-6 synthesis. J. Neuroinflammation 14:23. doi: 10.1186/s12974-017-0794-9 28122624 PMC5267445

[ref107] Patel C. A. Mukhtar M. Pomerantz R. J. (2000). Human immunodeficiency virus type 1 Vpr induces apoptosis in human neuronal cells. J. Virol. 74, 9717–9726. doi: 10.1128/jvi.74.20.9717-9726.2000 11000244, 11000244 PMC112404

[ref108] Pérez P. S. Romaniuk M. A. Duette G. A. Zhao Z. Huang Y. Martin-Jaular L. . (2019). Extracellular vesicles and chronic inflammation during HIV infection. J. Extracell. Vesicles 8:1687275. doi: 10.1080/20013078.2019.1687275 31998449 PMC6963413

[ref109] Pérez-Valero I. Ellis R. Heaton R. Deutsch R. Franklin D. Clifford D. B. . (2019). Cerebrospinal fluid viral escape in aviremic HIV-infected patients receiving antiretroviral therapy: prevalence, risk factors and neurocognitive effects. AIDS 33, 475–481. doi: 10.1097/QAD.0000000000002074 30702516 PMC6361539

[ref110] Planès R. Ben Haij N. Leghmari K. Serrero M. BenMohamed L. Bahraoui E. (2016). HIV-1 Tat protein activates both the MyD88 and TRIF pathways to induce tumor necrosis factor alpha and interleukin-10 in human monocytes. J. Virol. 90, 5886–5898. doi: 10.1128/JVI.00262-16 27053552 PMC4907244

[ref111] Polazzi E. Levi G. Minghetti L. (1999). Human immunodeficiency virus type 1 Tat protein stimulates inducible nitric oxide synthase expression and nitric oxide production in microglial cultures. J. Neuropathol. Exp. Neurol. 58, 825–831. doi: 10.1097/00005072-199908000-00005 10446807

[ref112] Potter M. C. Figuera-Losada M. Rojas C. Slusher B. S. (2013). Targeting the glutamatergic system for the treatment of HIV-associated neurocognitive disorders. J. Neuroimmune Pharmacol. 8, 594–607. doi: 10.1007/s11481-013-9442-z, 23553365 PMC3661915

[ref113] Ragin A. B. Wu Y. Gao Y. Keating S. Du H. Sammet C. . (2015). Brain alterations within the first 100 days of HIV infection. Ann. Clin. Transl. Neurol. 2, 12–21. doi: 10.1002/acn3.136 25642430 PMC4301670

[ref114] Raybuck J. D. Hargus N. J. Thayer S. A. (2017). A GluN2B-selective NMDAR antagonist reverses synapse loss and cognitive impairment produced by the HIV-1 protein Tat. J. Neurosci. 37, 7837–7847. doi: 10.1523/JNEUROSCI.0226-17.2017 28716964 PMC5559761

[ref115] Raymond A. D. Diaz P. Chevelon S. Agudelo M. Yndart-Arias A. Ding H. . (2016). Microglia-derived HIV Nef+ exosome impairment of the blood-brain barrier is treatable by nanomedicine-based delivery of Nef peptides. J. Neurovirol. 22, 129–139. doi: 10.1007/s13365-015-0397-0 26631079

[ref116] Rogers J. T. Morganti J. M. Bachstetter A. D. Hudson C. E. Peters M. M. Grimmig B. A. . (2011). CX3CR1 deficiency leads to impairment of hippocampal cognitive function and synaptic plasticity. J. Neurosci. 31, 16241–16250. doi: 10.1523/JNEUROSCI.3667-11.2011 22072675 PMC3236509

[ref117] Rossol M. Pierer M. Raulien N. Quandt D. Meusch U. Rothe K. . (2012). Extracellular Ca^2+^ is a danger signal activating the NLRP3 inflammasome through G protein-coupled calcium sensing receptors. Nat. Commun. 3:1329. doi: 10.1038/ncomms2339 23271661 PMC3535422

[ref118] Sanford R. Ances B. M. Meyerhoff D. J. Price R. W. Fuchs D. Zetterberg H. . (2018). Longitudinal trajectories of brain volume and cortical thickness in treated and untreated primary human immunodeficiency virus infection. Clin. Infect. Dis. 67, 1697–1704. doi: 10.1093/cid/ciy362 29697762 PMC6233681

[ref119] Schaffer Aguzzoli C. Ferreira P. C. L. Povala G. Ferrari-Souza J. P. Bellaver B. Soares Katz C. . (2023). Neuropsychiatric symptoms and microglial activation in patients with Alzheimer disease. JAMA Netw. Open 6:e2345175. doi: 10.1001/jamanetworkopen.2023.45175, 38010651 PMC10682836

[ref120] Schenck J. K. Karl M. T. Clarkson-Paredes C. Bastin A. Pushkarsky T. Brichacek B. . (2024). Extracellular vesicles produced by HIV-1 Nef-expressing cells induce myelin impairment and oligodendrocyte damage in the mouse central nervous system. J. Neuroinflammation 21:127. doi: 10.1186/s12974-024-03124-5 38741181 PMC11090814

[ref121] Self R. L. Mulholland P. J. Nath A. Harris B. R. Prendergast M. A. (2004). The human immunodeficiency virus type-1 transcription factor Tat produces elevations in intracellular Ca^2+^ that require function of an N-methyl-D-aspartate receptor polyamine-sensitive site. Brain Res. 995, 39–45. doi: 10.1016/j.brainres.2003.09.052 14644469

[ref122] Sfera A. Thomas K. G. Andronescu C. V. Jafri N. Sfera D. O. Sasannia S. . (2022). Bromodomains in human-immunodeficiency virus-associated neurocognitive disorders: a model of Ferroptosis-induced neurodegeneration. Front. Neurosci. 16:904816. doi: 10.3389/fnins.2022.904816 35645713 PMC9134113

[ref123] Shaw G. M. Hunter E. (2012). HIV transmission. Cold Spring Harb. Perspect. Med. 2:a006965. doi: 10.1101/cshperspect.a006965 23043157 PMC3543106

[ref124] Sheng W. S. Hu S. Hegg C. C. Thayer S. A. Peterson P. K. (2000). Activation of human microglial cells by HIV-1 gp41 and Tat proteins. Clin. Immunol. 96, 243–251. doi: 10.1006/clim.2000.4905 10964543

[ref125] Shiu C. Barbier E. Di Cello F. Choi H. J. Stins M. (2007). HIV-1 gp120 as well as alcohol affect blood-brain barrier permeability and stress fiber formation: involvement of reactive oxygen species. Alcohol. Clin. Exp. Res. 31, 130–137. doi: 10.1111/j.1530-0277.2006.00271.x 17207111

[ref126] Si Q. Kim M. O. Zhao M. L. Landau N. R. Goldstein H. Lee S. (2002). Vpr- and Nef-dependent induction of RANTES/CCL5 in microglial cells. Virology 301, 342–353. doi: 10.1006/viro.2002.1613 12359436

[ref127] Simons M. Nave K. A. (2016). Oligodendrocytes: myelination and axonal support. Cold Spring Harb. Perspect. Biol. 8:a020479. doi: 10.1101/cshperspect.a020479, 26101081 PMC4691794

[ref128] Simpson D. S. A. Oliver P. L. (2020). ROS generation in microglia: understanding oxidative stress and inflammation in neurodegenerative disease. Antioxidants 9:743. doi: 10.3390/antiox9080743 32823544 PMC7463655

[ref129] Singer E. J. Thames A. D. (2016). Neurobehavioral manifestations of HIV/AIDS: diagnosis and treatment. Neurol. Clin. 34, 33–53. doi: 10.1016/j.ncl.2015.08.003 26613994 PMC4663681

[ref130] Srichawla B. S. Kipkorir V. Manan M. R. Dhali A. Diebel S. Sawant T. . (2023). Stealth invaders: unraveling the mystery of neurotropic viruses and their elusive presence in cerebrospinal fluid – a comprehensive review. Ann. Med. Surg. 85, 2761–2766. doi: 10.1097/MS9.0000000000000736 PMC10289609 37363567

[ref131] Stoll F. M. Rudebeck P. H. (2024). Decision-making shapes dynamic inter-areal communication within macaque ventral frontal cortex. Curr. Biol. 34, 4526–4538.e5. doi: 10.1016/j.cub.2024.08.034 39293441 PMC11461104

[ref132] Tamargo J. A. Campa A. Martinez S. S. Li T. Sherman K. E. Zarini G. . (2021). Cognitive impairment among people who use heroin and fentanyl: findings from the Miami adult studies on HIV (MASH) cohort. J. Psychoactive Drugs 53, 215–223. doi: 10.1080/02791072.2020.1850946, 33225878 PMC8140063

[ref133] Tang P. Chong L. Li X. Liu Y. Liu P. Hou C. . (2014). Correlation between serum RANTES levels and the severity of Parkinson’s disease. Oxidative Med. Cell. Longev. 2014:208408. doi: 10.1155/2014/208408 PMC4283268 25587378

[ref134] Tang Z. Lu Y. Dong J. L. Wu W. Li J. (2024). The extracellular vesicles in HIV infection and progression: mechanisms, and theranostic implications. Front. Bioeng. Biotechnol. 12:1376455. doi: 10.3389/fbioe.2024.1376455 38655385 PMC11035885

[ref135] Thangaraj A. Chivero E. T. Tripathi A. Singh S. Niu F. Guo M. L. . (2020). HIV TAT-mediated microglial senescence: role of SIRT3-dependent mitochondrial oxidative stress. Redox Biol. 40:101843. doi: 10.1016/j.redox.2020.101843, 33385630 PMC7779826

[ref136] Thangaraj A. Periyasamy P. Liao K. Bendi V. S. Callen S. Pendyala G. . (2018). HIV-1 TAT-mediated microglial activation: role of mitochondrial dysfunction and defective mitophagy. Autophagy 14, 1596–1619. doi: 10.1080/15548627.2018.1476810 29966509 PMC6135576

[ref137] Timothy S. Malka S. Jesse K. Gabriela M. N. Conor L. (2021). Prefrontal deep projection neurons enable cognitive flexibility via persistent feedback monitoring. Cell 184, 2750–2766.e17. doi: 10.1016/j.cell.2021.03.047, 33861951 PMC8684294

[ref138] Triantafilou K. Ward C. J. K. Czubala M. Ferris R. G. Koppe E. Haffner C. . (2021). Differential recognition of HIV-stimulated IL-1β and IL-18 secretion through NLR and NAIP signalling in monocyte-derived macrophages. PLoS Pathog. 17:e1009417. doi: 10.1371/journal.ppat.1009417 33861800 PMC8109768

[ref139] Underwood J. Cole J. H. Caan M. De Francesco D. Leech R. van Zoest R. A. . (2017). Gray and white matter abnormalities in treated human immunodeficiency virus disease and their relationship to cognitive function. Clin. Infect. Dis. 65, 422–432. doi: 10.1093/cid/cix301 28387814 PMC5850629

[ref140] UpToDate (2026). Overview of the Neuropsychiatric Aspects of HIV Infection and AIDS – UpToDate. Available online at: https://www.uptodate.com/contents/overview-of-the-neuropsychiatric-aspects-of-hiv-infection-and-aids (Accessed May 4, 2026)

[ref141] Vastag Z. Fira-Mladinescu O. Rosca E. C. (2022). HIV-associated neurocognitive disorder (HAND): obstacles to early neuropsychological diagnosis. Int J Gen Med. 15, 4079–4090. doi: 10.2147/IJGM.S295859 35450033 PMC9017704

[ref142] Vilhardt F. Plastre O. Sawada M. Suzuki K. Wiznerowicz M. Kiyokawa E. . (2002). The HIV-1 Nef protein and phagocyte NADPH oxidase activation. J. Biol. Chem. 277, 42136–42143. doi: 10.1074/jbc.M200862200 12207012

[ref143] Walsh K. A. Megyesi J. F. Wilson J. X. Crukley J. Laubach V. E. Hammond R. R. (2004). Antioxidant protection from HIV-1 gp120-induced neuroglial toxicity. J. Neuroinflammation 1:8. doi: 10.1186/1742-2094-1-8 15285794 PMC483061

[ref144] Walsh J. G. Reinke S. N. Mamik M. K. McKenzie B. A. Maingat F. Branton W. G. . (2014). Rapid inflammasome activation in microglia contributes to brain disease in HIV/AIDS. Retrovirology 11:35. doi: 10.1186/1742-4690-11-35, 24886384 PMC4038111

[ref145] Wang Y. Liu M. Lu Q. Farrell M. Lappin J. M. Shi J. . (2020). Global prevalence and burden of HIV-associated neurocognitive disorder: a meta-analysis. Neurology 95, e2610–e2621. doi: 10.1212/WNL.0000000000010752 32887786

[ref146] Wang Z. Pekarskaya O. Bencheikh M. Chao W. Gelbard H. A. Ghorpade A. . (2003). Reduced expression of glutamate transporter EAAT2 and impaired glutamate transport in human primary astrocytes exposed to HIV-1 or gp120. Virology 312, 60–73. doi: 10.1016/S0042-6822(03)00181-8, 12890621

[ref147] Wang Y. Santerre M. Tempera I. Martin K. Mukerjee R. Sawaya B. E. (2017). HIV-1 Vpr disrupts mitochondria axonal transport and accelerates neuronal aging. Neuropharmacology 117, 364–375. doi: 10.1016/j.neuropharm.2017.02.008 28212984 PMC5397298

[ref148] Watson Z. T. Valdebenito-Silva S. Spurgat M. Ru W. Zheng J. Maurelli M. K. . (2025). Differential selectivity of microglia and astrocytes in HIV-1 gp120-induced synaptic pruning. Brain:awaf429. doi: 10.1093/brain/awaf429, 41234167 PMC13285724

[ref149] World Health Organization. (2026). HIV global situation and trends. Available online at: https://www.who.int/data/gho/data/themes/hiv-aids (Accessed May 1, 2026)

[ref150] Wu D. Chen Q. Chen X. Han F. Chen Z. Wang Y. (2023). The blood–brain barrier: structure, regulation and drug delivery. Sig Transduct Target Ther. 8:217. doi: 10.1038/s41392-023-01481-w, 37231000 PMC10212980

[ref151] Wu Y. Storey P. Cohen B. A. Epstein L. G. Edelman R. R. Ragin A. B. (2006). Diffusion alterations in corpus callosum of patients with HIV. AJNR Am. J. Neuroradiol. 27, 656–660., 16552012 PMC2901995

[ref152] Xu Z. Chen Z. m. Wu X. Zhang L. Cao Y. Zhou P. (2020). Distinct molecular mechanisms underlying potassium efflux for NLRP3 inflammasome activation. Front. Immunol. 11:609441. doi: 10.3389/fimmu.2020.609441, 33424864 PMC7793832

[ref153] Yadav-Samudrala B. J. Yadav A. P. Patel R. P. Fitting S. (2025). HIV-1 tat protein alters medial prefrontal cortex neuronal activity and recognition memory. iScience. 28:112075. doi: 10.1016/j.isci.2025.112075, 40160418 PMC11952812

[ref154] Yang X. Han P. Li M. Xue Y. Yu X. Jiang M. . (2025). HIV-1 Tat mediates microglial NLRP3 inflammasome activation and neurotoxicity by inducing cytosolic mtDNA stress. Int. J. Biol. Macromol. 318:145093. doi: 10.1016/j.ijbiomac.2025.145093, 40494474

[ref155] Yates J. R. (2024). Aberrant glutamatergic systems underlying impulsive behaviors: insights from clinical and preclinical research. Prog. Neuro-Psychopharmacol. Biol. Psychiatry 135:111107. doi: 10.1016/j.pnpbp.2024.111107 PMC11409449 39098647

[ref156] Ye X. Zhang Y. Xu Q. Zheng H. Wu X. Qiu J. . (2017). HIV-1 Tat inhibits EAAT-2 through AEG-1 upregulation in models of HIV-associated neurocognitive disorder. Oncotarget 8, 39922–39934. doi: 10.18632/oncotarget.16485 28404980 PMC5503662

[ref157] Yuan N. Y. Kaul M. (2021). Beneficial and adverse effects of cART affect neurocognitive function in HIV-1 infection: balancing viral suppression against neuronal stress and injury. J. Neuroimmune Pharmacol. 16, 90–112. doi: 10.1007/s11481-019-09868-9 31385157 PMC7233291

[ref158] Zanetti L. Regoni M. Ratti E. Valtorta F. Sassone J. (2021). Presynaptic AMPA receptors in health and disease. Cells 10:2260. doi: 10.3390/cells10092260 34571906 PMC8470629

[ref159] Zhao J. Bu F. Wu H. He J. Liu J. (2026). Neuroinflammation and NeuroHIV: understanding the role of HIV-1 related factors in microglial activation. Transl. Psychiatry 16:194. doi: 10.1038/s41398-026-03941-7, 41856995 PMC13039484

[ref160] Zheng D. Liwinski T. Elinav E. (2020). Inflammasome activation and regulation: toward a better understanding of complex mechanisms. Cell Discov. 6:36. doi: 10.1038/s41421-020-0167-x, 32550001 PMC7280307

[ref161] Zou S. Fuss B. Fitting S. Hahn Y. K. Hauser K. F. Knapp P. E. (2015). Oligodendrocytes are targets of HIV-1 Tat: NMDA and AMPA receptor-mediated effects on survival and development. J. Neurosci. 35, 11384–11398. doi: 10.1523/JNEUROSCI.4740-14.2015 26269645 PMC4532766

