# Supplementary material for: The prefrontal cortex as a target of HIV-1 neurotoxicity: molecular mechanisms of viral protein-mediated neurodegeneration and executive dysfunction
Source: Front Cell Neurosci. 2026 Aug 18;20:1899622. doi: 10.3389/fncel.2026.1899622 (PMC13531410; doi:10.3389/fncel.2026.1899622)
Supplement: Supplementary file 1 [file Data_Sheet_1.DOCX]

**Supplement File 1. Prompts used for generative AI Figure Labs to create figures.**

**Figure 1 uploaded information:**

Specific signs and symptoms that often manifest as the condition progresses through the symptomatic stages (MND and HAD):

Cognitive Signs: These include decreased information processing speed (the most common dysfunction), executive dysfunction, amnesia, short-term memory loss, a decline in rational abilities, and problems with focus and attention.

Behavioral & Emotional Symptoms: Patients may experience depression, lethargy, anxiety, anger, a reduction in motivation, or a decrease in impulsiveness.

Motor Signs: Physical manifestations include slower actions, shivering, convulsions, clumsiness, poor coordination, and overactive deep tendon reflexes. A hallmark sign is the inability to perform fine-finger operations.

Diagnostic evaluations for these signs typically assess at least five domains, including attention-information processing, language, abstraction/executive functions, complex perceptual motor skills, and memory.

| HAND Type | Neurocognitive Status (Objective Signs) | Functional Status (Clinical Symptoms) |
| --- | --- | --- |
| Asymptomatic Neurocognitive Impairment (ANI) | Cognitive impairment in ≥2 domains (functioning at least 1 SD below normal). | No observable interference or symptoms in daily functioning. |
| Mild Neurocognitive Disorder (MND) | Cognitive impairment in ≥2 domains (functioning at least 1 SD below normal). | Minor interference in daily life. Patients may report reduced mental perception or incompetence at work, often supported by observations from others. |
| HIV-Associated Dementia (HAD) | Severe cognitive impairment in ≥2 domains (functioning at least 2 SD below normal). | Noticeable and significant impairment in everyday living. May manifest as severe cognitive, behavioral, and motor failure |

**Figure 1 image generation prompt:**

1. Create an image showing the different signs and symptoms of the different spectrums of HAND, including a panel on the diagnostic criteria.
2. Increase this image to 2K resolution with sharper and clearer details

**Figure 2 prompt:**

- Tat is able to increase intracellular Ca^2+^ within microglia, which is a known danger-associated molecular pattern (DAMP) signal for inflammasome activation Secondly, Tat was recently discovered to cause neurotoxicity by inducing mitochondrial stress and increase in ROS, leading to leakage of mitochondrial DNA (mtDNA) into the cytoplasm, another inflammasome activation DAMP signal.
- Gp120 can cause the priming and subsequent activation of the NLRP3 inflammasome, albeit by a different mechanism. When gp120 binds to CXCR4 on microglial cells, receptor signaling activates NF-κB, which in turn upregulates the transcription of NLRP3 and pro-IL-1β. This primes the cell for inflammasome assembly. Gp120 can also trigger K^+^ efflux when binding to the cell co-receptor, causing the direct activation of the NLRP3 inflammasome
- Nef is also responsible for the activation of the NLRP3 inflammasome in microglia. Research shows that once macrophages were exposed to Nef in exosomes, cholesterol efflux was reduced, which resulted in an increase in lipid rafts in the membrane. This altered the localization of TLR4, activating signaling kinases, and subsequent primed and activated the NLRP3 inflammasome, leading to a strong proinflammatory response.
- Vpr has been detected in the CSF of patients with HIV, and in supernatants of HIV-infected human microglia. This protein was shown to directly promote the production of IL-1β in primary human microglia, in an NLRP3-dependent manner, activating the assembly of the inflammasome and subsequently caused reduced cell viability due to pyroptosis. This study also observed that transgenic mice who expressed Vpr in the brain developed neurotoxicity, with resulting neurodegeneration typically associated with HAND. The ability of Vpr to damage mitochondria and cause oxidative stress provides the DAMP signal that initiates NLRP3 activation.
- Vpu can prime the inflammasome pathway via binding to TLR4, which results in transcription of pro-IL-1β and NLRP3. There is also evidence that Vpu interacts with cell membrane K^+^ channels, causing K^+^ efflux. This reduction in cytosolic K^+^ is a trigger (DAMP) for NLRP3 activation, by causing a conformational change and assembly of the multi-protein inflammasome unit.
- It should be noted that recent evidence has shown that gp41 can directly activate the NAIP/NLRC4 inflammasome without any priming, leading to secretion of active IL-18. This is due to the ability of gp41 to bind to the pathway’s sensor protein, NAIP, in macrophages.
- Do not label the image, and figure legend is not necessary
- Increase this image to 2K resolution with sharper and clearer details
- After image generation, author added individual subsection letters A-F, as well as added The HIV protein name only to label each subsection

**Figure 3 (Ferroptosis)**

- Tat and gp120 have been shown to increase the pool of free redox-reactive intracellular Fe^2+^ by causing endolysosomal deacidification during initial viral infection, which causes efflux of free iron into the cytoplasm. This increases the opportunity for Fenton reactions to attack lipids. Secondly, Tat, and general proinflammatory activation of microglia by HIV, release lipid droplets via lipid dysregulation. These droplets help traffic and incorporate PUFAs into the cell membrane, which act as the prime target for peroxidation. Therefore, the overall target lipid pool increases. Thirdly, Tat suppresses an important antioxidant enzyme, GPX4, by inhibiting cystine import (System Xc^-^) and GSH synthesis, preventing GPX4 function. This results in unchecked accumulation of even more lipid peroxides, as Fe^2+^ attacks the PUFAs in the plasma membrane. Fourthly, Tat specifically activates Acyl-CoA Synthase Long-Chain Family Member 4 (ACSL4), a “Master Regulator” of ferroptosis activation. When ACSL4 is active, PUFAs are enriched in the cell membrane, further amplifying available lipids to be peroxidized to levels that trigger ferroptosis. Fifthly, as discussed above, Tat activates NADPH oxidase in microglia via NF-κB and MAPK signaling, which causes mitochondrial damage and ROS leakage, which increases lipid peroxidation. Finally, Tat increases intracellular Ca^2+^ within microglia, a known DAMP trigger for ferroptosis, as it increases intracellular ROS.
- Do not label the image, and figure legend is not necessary
- Keep same image but remove the 2nd gp120 and the receptor it is binding to on the left, as it is duplicating information
- Increase this image to 2K resolution with sharper and clearer details
- After image generation, author added individual subsection numbers (1-8)

**Figure 4 (Glutamatergic Dysregulation)**

- Panel A: Tat binds to NMDA Receptors (NMDAR), triggering calcium influx. Tat also interacts with AMPA Receptors (AMPARs) on oligodendrocytes that causes calcium influx and myelin sheath disruption. Tat can also bind to mGluRs which stimulates exocytosis of additional glutamate from nerve endings. Tat directly downregulates EAAT2 receptor expression and function on astrocytes, causing them to be less-efficient at clearing glutamate. Tat promotes the upregulation of the cystine-glutamate antiporter in microglia and astrocytes, leading to even more glutamate release into the extracellular space.
- Panel B: Human astrocytes exposed to gp120 showed reduced expression of EAAT2 receptor. Gp120 has been shown to reduce the expression of glutamine synthase in astrocytes, which is responsible for converting glutamate into glutamine. gp120 has been shown to directly interact with NMDARs, causing neuronal injury in a similar mechanism to Tat.
- Do not label the image
- Make a figure legend labeling: NMDAR and mGluR receptors on neurons, AMPAR receptor on oligodendrocytes, EAAT2 receptor on astrocytes, and cystine-glutamate antiporters on microglia and astrocytes; also include in the legend glutamate, Tat, gp120, glutamine synthase, so readers can clearly understand the image
- Keep the image but remove all words/text. Just have the images
- Increase this image to 2K resolution with sharper and clearer details
- After image generation, author added individual subsections (A and B), and labels for each component as appropriate
